# Coding and non-coding gene regulatory networks underlie the immune response in liver cirrhosis

**DOI:** 10.1371/journal.pone.0174142

**Published:** 2017-03-29

**Authors:** Bo Gao, Xueming Zhang, Yongming Huang, Zhengpeng Yang, Yuguo Zhang, Weihui Zhang, Zu-hua Gao, Dongbo Xue

**Affiliations:** 1 Department of General Surgery, The First Affiliated Hospital of Harbin Medical University, Harbin, China; 2 Department of Pathology, The Research Institute of McGill University Health Centre, Montreal, Quebec, Canada; 3 Department of Traditional and Western Medical Hepatology, The Third Hospital of Hebei Medical University, Shijiazhuang, China; National Institutes of Health, UNITED STATES

## Abstract

Liver cirrhosis is recognized as being the consequence of immune-mediated hepatocyte damage and repair processes. However, the regulation of these immune responses underlying liver cirrhosis has not been elucidated. In this study, we used GEO datasets and bioinformatics methods to established coding and non-coding gene regulatory networks including transcription factor-/lncRNA-microRNA-mRNA, and competing endogenous RNA interaction networks. Our results identified 2224 mRNAs, 70 lncRNAs and 46 microRNAs were differentially expressed in liver cirrhosis. The transcription factor -/lncRNA- microRNA-mRNA network we uncovered that results in immune-mediated liver cirrhosis is comprised of 5 core microRNAs (e.g., miR-203; miR-219-5p), 3 transcription factors (i.e., FOXP3, ETS1 and FOS) and 7 lncRNAs (e.g., ENTS00000671336, ENST00000575137). The competing endogenous RNA interaction network we identified includes a complex immune response regulatory subnetwork that controls the entire liver cirrhosis network. Additionally, we found 10 overlapping GO terms shared by both liver cirrhosis and hepatocellular carcinoma including “immune response” as well. Interestingly, the overlapping differentially expressed genes in liver cirrhosis and hepatocellular carcinoma were enriched in immune response-related functional terms. In summary, a complex gene regulatory network underlying immune response processes may play an important role in the development and progression of liver cirrhosis, and its development into hepatocellular carcinoma.

## Introduction

Liver cirrhosis is a common end point for various chronic liver diseases including chronic viral hepatitis due to hepatitis B or hepatitis C viral infections, alcoholic or nonalcoholic fatty liver disease, autoimmune hepatitis, biliary disorders and inherited metabolic defects[1]. Regardless of specific etiology, immune-mediated liver damage in each of these diseases eventually leads to liver cirrhosis[2]. Histologically, immune-mediated liver damage presents as a loss of hepatocytes and the accumulation of lymphocytes, macrophages, and stromal cells, which interact in a paracrine manner through the secretion of proteins including chemokines and cytokines. Therefore, elucidating the gene regulatory network for the immunological processes that lead to liver cirrhosis could help to characterize its pathogenesis to develop more effective therapies.

Complications of liver cirrhosis include liver dysfunction, portal hypertension and the development of hepatocellular carcinoma (HCC). Epidemiological studies have shown that cirrhosis is the main cause of HCC, and the progression of HCC in some cases follows the three steps of “hepatitis—cirrhosis—HCC”[3]. Therefore, the identification of the genetic regulatory networks that underlie liver cirrhosis and HCC following liver cirrhosis is crucial in the development of more effective strategies that prevent the progression from liver cirrhosis to HCC.

Numerous studies have shown that the gene regulatory relationships are extensive in liver cirrhosis and that they play essential roles in its development and progression. Sekiya et al. found that the overexpression of miR-29b could inhibit the expression of the FOS TF, thereby suppressing the activation of hepatic stellate cells (HSCs) in liver cirrhosis and ultimately facilitating its development[4]. Moreover, various regulatory relationships exist between coding and non-coding genes in complex gene regulatory networks. For instance, coding genes act as competitive endogenous RNAs (ceRNAs) for one another, microRNAs (miRNAs) bind to sites in the 3'-termini of their target genes to regulate gene expression, miRNAs and transcription factors (TFs) jointly regulate target gene expression through feedforward and feedback loops, and long non-coding RNAs (lncRNAs) act as ceRNAs to regulate target gene expression[5–7]. The analysis of such complex genetic interactions through traditional research methods and decomposition analysis is challenging. Gene regulatory network analysis is a stable and dynamic hierarchical system model that is increasingly used to study various diseases. This model can combine high-throughput gene expression data, gene interaction information, and relevant analysis and calculation methods[8, 9]. From a macro perspective, the gene regulatory network underlying the development and progression of liver cirrhosis is comprised of many individual regulatory relationships. Integrating these relationships using gene network analysis enables a clear and systematic examination of liver cirrhosis.

In the present study, we performed a gene regulatory network analysis of differentially expressed (DE) coding and non-coding genes in liver cirrhosis using bioinformatics. We screened the important miRNAs, lncRNAs, TFs, and their respective gene networks that were involved in the progression of liver cirrhosis and specifically analyzed the subnetworks of functions related to the immune response in liver cirrhosis. Additionally, we used DE genes in HCC to screen DE genes and gene ontology (GO) terms that overlapped in the liver cirrhosis and HCC networks. This study provides insight into the pathogenesis of liver cirrhosis and HCC following liver cirrhosis.

## Materials and methods

### Data sources

The lncRNA, mRNA, and miRNA data used in the study were retrieved from the GSE54238 and GSE63046 datasets in the GEO database (http://www.ncbi.nlm.nih.gov/gds/). From dataset GSE54238, we selected 10 normal liver tissue samples as the control group (NL group) and 10 liver cirrhosis tissue samples from patients with liver cirrhosis as the liver cirrhosis group to screen DE lncRNAs and mRNAs in liver cirrhosis. The samples with viral liver cirrhosis were used to identify the DE genes. From dataset GSE63046, we selected 9 normal liver tissue samples as the control group (NL group) and 15 liver cirrhosis tissue samples from patients with liver cirrhosis as the liver cirrhosis group to screen miRNAs in liver cirrhosis.

### Data processing

The GSE54238 dataset consisted of Arraystar microarray expression data. We extracted the original signals of the probes from the microarray samples separately, and then used the Limma package to preprocess the microarray gene expression data. The preprocessing procedure included robust multi-array average (RMA) background correction, quantile normalization, and probe summarization to ultimately obtain the gene expression matrix. Because the identification (ID) of lncRNAs recorded in the GSE54238 dataset was unofficial, we separately extracted all lncRNA information recorded on this set of microarrays and matched it with the official ID. After unifying the different versions of the annotated genome, we compared the transcript region for all lncRNAs recorded in the microarray and the official transcript region recorded in the Ensemble database. We extracted lncRNAs with more than 90% overlapping regions and matched the corresponding Ensemble ID. During ID matching, the probe signals were averaged if more than one lncRNA recorded in the microarray was matched to a single official Ensemble sequence. The lncRNA record was omitted if the lncRNA recorded in the microarray was not matched to any official Ensemble sequence.

We processed the second-generation sequencing dataset GSE63046. First, we removed linker sequences from all small RNA-seq data and retained all original sequences at least 16 bp in length. Next, we screened the sequencing quality of these sequences and set the definition of high-quality bases (Phred score ≥20) in each read to at least 90% of the total length of the read and a base uncertainty of no more than 5%. After the completion of the quality control and data cleaning of the raw data, we removed sequences from the non-coding RNAs (e.g., rRNA, tRNA, and snRNA) in the sequencing results according to the information recorded in the Rfam database. Then, we aligned the filtered sequences and performed quantitative calculations of the miRNA using miRDeep2 combined with the known human miRNAs recorded in miRbase. The quantitation of each miRNA was performed using the TPM (transcripts per million) method: TPM = number of miRNA reads aligned × 10^6^/total number of reads. Finally, we excluded miRNA records with zero expression levels in all samples. For miRNAs with expression in topical samples, we defined the TPM as 0.0001 to ensure subsequent differential analysis.

### Differential expression analysis of mRNAs, lncRNAs and miRNAs

We used the Bioconductor Limma package to identify DE genes. According to the experimental design, we selected the unpaired moderated t-test to assess the significance level of DE lncRNAs and mRNAs between liver cirrhosis and normal groups. The adjusted P value (false discovery rate, FDR) of each gene was calculated using the Benjamini Hochberg (BH) method. For each significant DE gene, we set the significance level of differential expression to FDR < 0.05 and fold change ≥ 2 or ≤ 0.5. For the differential miRNA expression analysis, no significantly different results were obtained with an FDR < 0.05 and fold change ≥ 2 or ≤ 0.5. We adjusted the screening threshold for the differential miRNA expression analysis and selected significant DE miRNAs with p values less than 0.05 from the moderated t-test and fold changes ≥ 2 or ≤ 0.5.

### Construction of the TF—miRNA—mRNA regulatory network

We extracted significant DE miRNAs and mRNAs according to the results of the differential expression analysis and then constructed the TF—miRNA—mRNA regulatory network using a combination of TF-binding site data and miRNA target gene data. Based on the ENCODE database, we removed ChIP-seq data corresponding to histone modifications, histone modifying enzymes, CTCF insulators, and the enhancer-binding protein P300 recorded in the database[10]. We retained all TF binding site data on chromosomes in the database and included all TF binding site data in the downstream analysis. We also searched TF-binding sites that potentially existed in the promoter regions of the DE miRNAs. Here, we defined the scope of the promoter region as between 1500 bp upstream and 500 bp downstream of the transcription start site of each gene. During the analysis, a TF—miRNA pair was generated if there were known TF-binding sites in the promoter region of the miRNA. The DE miRNA and DE mRNA pairs were generated based the TargetScan database (http://www.targetscan.org). As required by the subsequent ceRNA analysis, we selected DE miRNA—mRNA pairs without the constraint that the regulatory directions of the miRNA and mRNA must be different. Based on the results, we constructed and visualized the TF—miRNA—mRNA regulatory network using the Cytoscape[11]. In the final network, the node color of the genes was set to different gradients according to the p value and the node size was set according to the degree.

### Construction of the lncRNA—miRNA—mRNA regulatory network

According to the results of the differential expression analysis, we extracted significant DE lncRNAs, miRNAs, and mRNAs to construct the lncRNA—miRNA—mRNA regulatory network. DE lncRNA and DE miRNA pairs were generated based on the lnCeDB database [12]. DE miRNA and DE mRNA pairs were generated based on the TargetScan database. When screening the DE miRNA—mRNA pairs, we did not use the constraint that the regulatory directions of the miRNA and mRNA must be different. Based on the results, we used Cytoscape to construct the lncRNA—miRNA—mRNA regulatory network. The node color of the genes was set to different gradients according to the p value and the node size was set according to the degree.

### Construction of the protein-coding ceRNA network

According to the results of the differential expression analysis, we extracted significant mRNAs to construct the protein-coding ceRNA network. All DE mRNAs that were regulated by the same miRNA were screened based on the TargetScan database. DE gene pairs were screened using the String database in combination with the results of the miRNA—mRNA analysis. Briefly, we searched other protein-coding genes that could interact with specific genes and generated the PPI module using the information provided by the String database (http://www.string-db.org). We only selected information on PPI pairs obtained by experimental verification, text mining, co-expression analysis or relevant records in the database as the input data for module construction. The PPI pairs included in the analysis contained at least one DE gene. The PPI network of the protein-coding ceRNA network was constructed according to the PPI results. In the network, the nodes were set to various sizes according to their degree.

### Screening of DE mRNAs in HCC

Based on the dataset GSE54238, we identified DE mRNAs in HCC from 10 liver cirrhosis samples and 10 early HCC samples. We selected the unpaired moderated t-test to assess the significance level of DE mRNAs between HCC and liver cirrhosis groups. The FDR of each gene was calculated using the Benjamini Hochberg (BH) method. For each significant DE gene, we set the significance level of differential expression to FDR < 0.05 and fold change ≥ 2 or ≤ 0.5. After that, based on DE mRNAs in liver cirrhosis, overlapping DE mRNAs were identified in liver cirrhosis and HCC and visualized in a Venn diagram.

### GO analysis

The gene ontology (GO) categories were derived from the Database for Annotation, Visualization and Integrated Discovery (DAVID, https://david.ncifcrf.gov/). A hypergeometric distribution test and FDR were used to identify significant DE GO terms with a p-value cut-off of 0.05.

## Results

### DE mRNA, miRNA and lncRNA in liver cirrhosis

**Fig 1:**
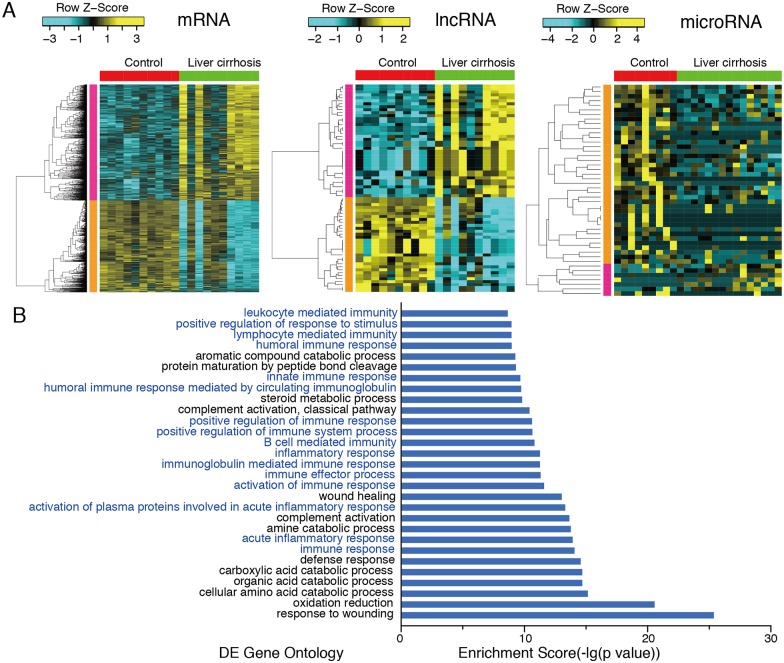
Identification and functional enrichment of DE genes in liver cirrhosis. (A) Cluster analysis heatmap of DE mRNA, lncRNA and microRNA in liver cirrhosis. 2224 DE mRNAs, 46 DE miRNAs and 70 DE lncRNAs were identified in liver cirrhosis. (B) GO analysis of DE gene in liver cirrhosis using the DAVID database. The bar plot shows the enrichment scores (-log FDR value) of the significant GO terms. 16 of the 30 GO terms with the highest statistical significance are immune response-related.

Based on the dataset GSE54238, we identified 2224 DE mRNAs and 70 DE lncRNAs in liver cirrhosis. Among them, 1239 mRNAs and 70 lncRNAs were upregulated; 985 mRNAs and 32 lncRNAs were downregulated. Based on the dataset GSE63046, we identified 46 DE miRNAs, including 6 upregulated and 40 downregulated miRNAs in liver cirrhosis. (S1 Table) Finally, we built a heatmap of these DE mRNAs, miRNAs and lncRNAs (Fig 1A).

### GO analysis of DE genes

GO analysis of DE mRNAs was determined using the DAVID database. There were 16 immune response-related GO terms in the 30 GO terms with the highest statistical significance including as “immune response”, “acute inflammatory response”, and "activation of immune response”, etc. A GO term with a false discovery rate (FDR) <0.05 was considered statistically significant (Fig 1B, S2 Table).

### TF—miRNA—mRNA regulatory network in liver cirrhosis

**Fig 2:**
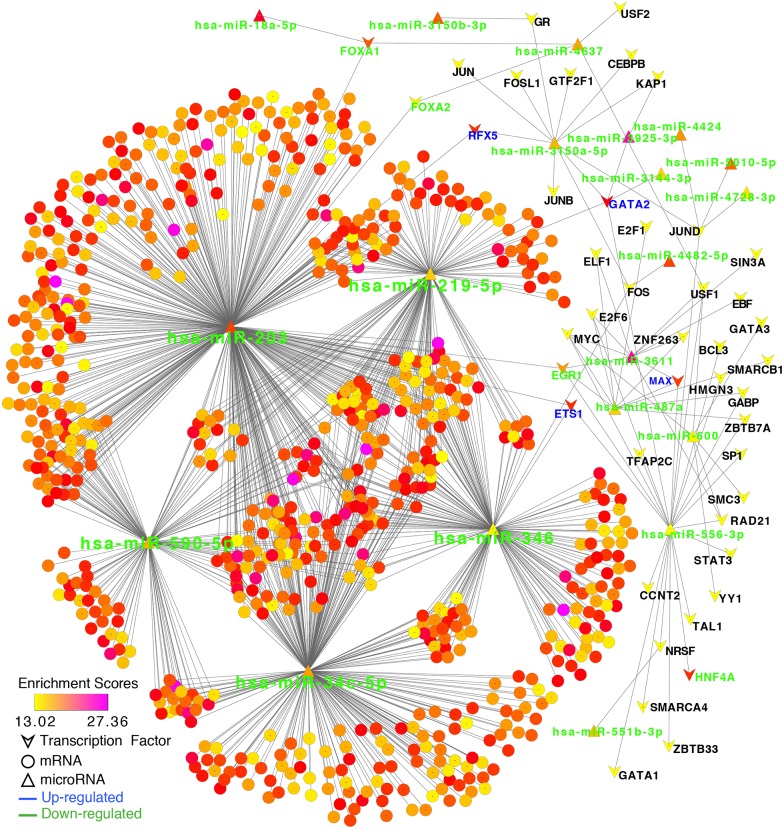
TF-miRNA-mRNA regulatory network in liver cirrhosis. Based on the TargetScan database and DE genes, we established a regulatory network including 19 DE miRNAs, 49 DE TFs and 745 DE mRNAs. The node size represents the degree of the node itself. The node color represents the enrichment score (-log p value).

We generated a total of 1293 miRNA—mRNA pairs using the TargetScan database in combination with the results of the differential miRNA and mRNA expression analyses. The miRNA—mRNA pairs included five DE miRNAs and 745 DE mRNAs. All five DE miRNAs were significantly downregulated in the liver cirrhosis group. Next, we searched all known TF binding sites in the promoter regions of all DE miRNAs using the ENCODE database and generated 80 TF—DE miRNA pairs, including 19 DE miRNAs and 49 TFs. Notably, all 19 miRNAs were significantly downregulated in the liver cirrhosis group and the known TF binding sites were not found in the promoter regions of any upregulated miRNAs. We constructed the TF—miRNA—mRNA regulatory network using a combination of data obtained from the TF—miRNA and miRNA—mRNA pairs. The TF—miRNA—mRNA regulatory network was visualized through using Cytoscape software (S1 File). In the regulatory network, the node size represents the degree of the node itself (i.e., the number of neighboring nodes directly connected to the node, which is an indicator of the importance of the node in the network). The node color represents the enrichment score (-log p value). Nodes with a high degree and enrichment score play important roles in the regulatory network (Fig 2). In total, we found five important miRNAs in the network, including has-miR-203(degree = 457), has-miR-34c-5p(degree = 314), has-miR-346(degree = 201), has-miR-219-5p(degree = 172), and has-miR-590-5p(degree = 150). We also obtained eight important TFs with relatively high degrees and enrichment scores that exhibited regulatory effects on the five miRNAs. The upregulated TFs included ETS1, FOS, FOXP3, RFX5, GATA2 and the downregulated TFs included EGR1, FOXA1, FOXA2. Moreover, we identified the top five miRNAs showing the most interactions with TFs in liver cirrhosis, including has-miR-556-3p(degree = 22), hsa-miR-3150a-5p(degree = 11), hsa-miR-3611(degree = 10), hsa-miR-487a(degree = 10), and hsa-miR-600(degree = 6).

### lncRNA—miRNA—mRNA regulatory network in liver cirrhosis

**Fig 3:**
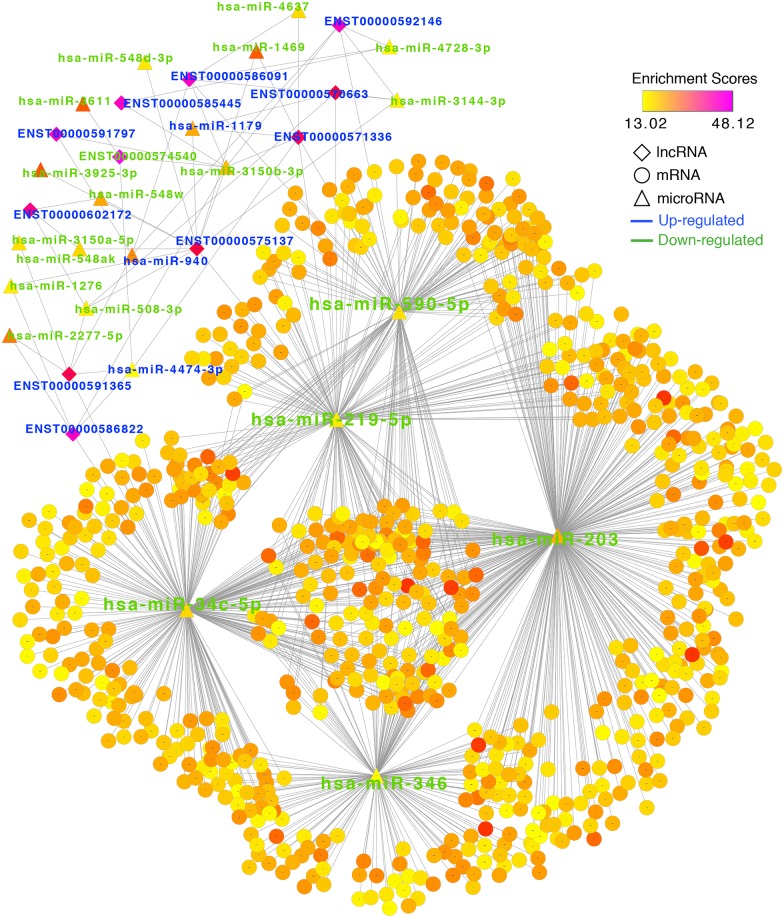
lncRNA-miRNA-mRNA regulatory network in liver cirrhosis. Based on the lnCeDB database and DE genes, we predicted miRNA-lncRNA pairs from 20 DE miRNAs and 11 DE lncRNAs. Next, we constructed the lncRNA—miRNA—mRNA regulatory network using a combination of data obtained from the miRNA—lncRNA and miRNA—mRNA pairs. The node size represents the degree of the node itself. The node color represents the enrichment score (-log p value).

In total, we generated 54 miRNA—lncRNA pairs using the lnCeDB database combined with the results of the differential miRNA and lncRNA expression analyses. The miRNA-lncRNA pairs included 20 DE miRNAs and 11 DE lncRNAs. Next, we constructed the lncRNA—miRNA—mRNA regulatory network using a combination of data obtained from the miRNA—lncRNA and miRNA—mRNA pairs (Fig 3). The lncRNA—miRNA—mRNA regulatory network was visualized through using Cytoscape software (S2 File). The lncRNA network and the TF network were generated using the same miRNA—mRNA pairs. We obtained five important miRNAs that were consistent with the results derived from the TF network. Additionally, we identified seven lncRNAs with relatively high degrees and enrichment scores that showed regulatory relationships with the five important miRNAs. These lncRNAs included ENST00000575137, ENST00000592146, ENST00000585445, ENST00000571336, ENST00000591365, ENST00000586822, ENST00000592146, ENST00000571336, ENST00000570663. It is worth noting that all of the important lncRNAs except ENST00000574540 were highly expressed in the liver cirrhosis group. ENST00000574540 showed low expression in the entire network. According to the ceRNA theory, these lncRNAs and coding genes in the network can competitively bind to the five important miRNAs to regulate the expression of the coding genes. Moreover, two lncRNAs that can bind to the same miRNA will act as ceRNAs to mutually regulate one another. We found that hsa-miR-3150b-3p could bind to most of the lncRNAs in the liver cirrhosis gene regulatory network.

### Immune response—Related gene regulatory subnetwork in liver cirrhosis

**Fig 4:**
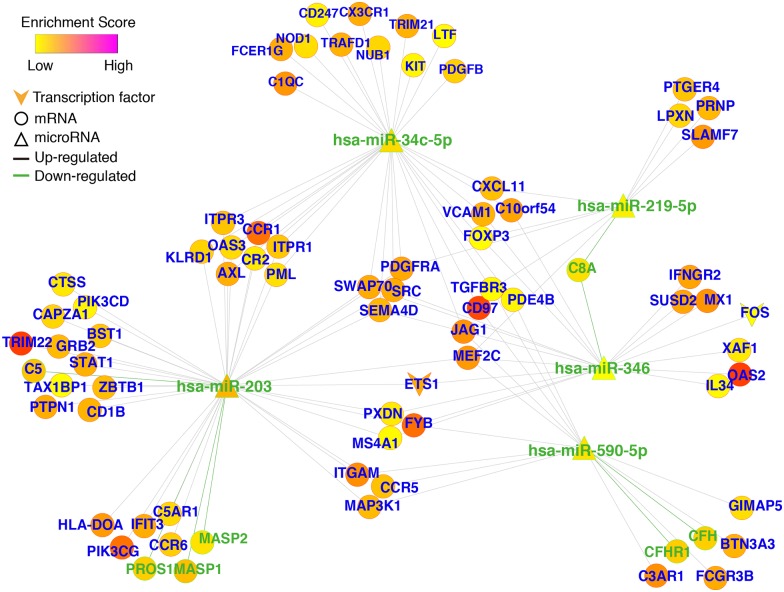
TF-miRNA-mRNA regulatory network underlying the immune response in liver cirrhosis. The subnetwork revealed 69 upregulated mRNAs, 7 downregulated mRNAs, 2 upregulated TFs (ETS1 and FOS) and 5 downregulated miRNAs.

Based on the DE mRNAs enriched in the immune response-related GO terms and the TF- and lncRNA-miRNA-mRNA networks in liver cirrhosis, we screened immune response—related TF- and lncRNA-miRNA-mRNA subnetworks. Cytoscape software was used to visualize and analyze the network (S3 File). The immune response-related TF-miRNA-mRNA subnetwork analyses revealed 69 upregulated mRNAs, 7 downregulated mRNAs, 2 upregulated TFs (ETS1 and FOS) and 5 downregulated miRNAs (Fig 4). Among them, ETS1 interacted with has-miR-203 and has-miR-346 and FOS interacted with has-miR-346. Five important miRNAs are consistent with DE miRNAs in the main network.

**Fig 5:**
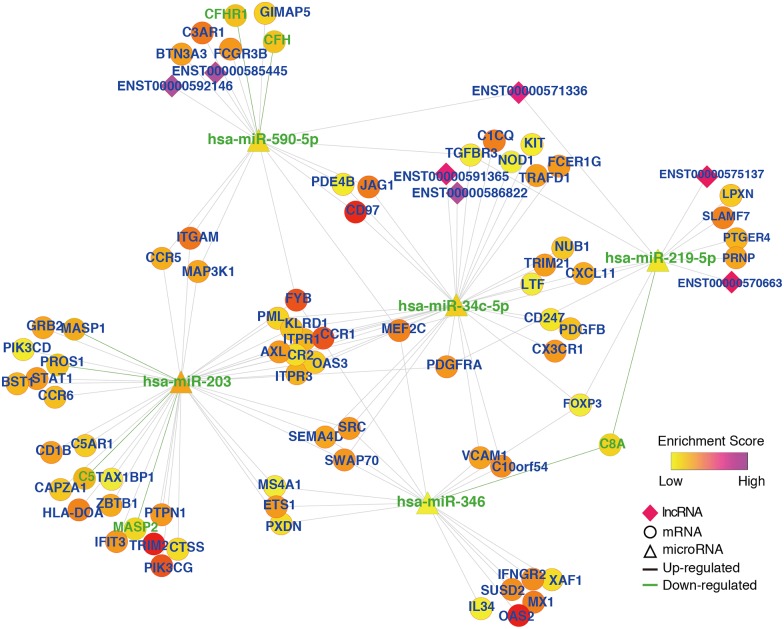
lncRNA-microRNA-mRNA regulatory network underlying the immune response in liver cirrhosis. 7 upregulated lncRNAs were revealed in the sub-network. The node size represents the degree of the node itself. The node color represents the enrichment score (-log p value).

Cytoscape software was used to visualize and analyze the immune response-related lncRNA-miRNA-mRNA subnetwork (S4 File). In the subnetwork, 7 upregulated lncRNAs were revealed including ENST00000571336, ENST00000592146, ENST00000585445, ENST00000591365, ENST00000586822, ENST00000575137, and ENST00000570663 (Fig 5). Among them, only ENST00000571336 had two interacting miRNAs (has-miR-590-5p和has-miR-219-5p), the other 6 lncRNAs had a single interacting miRNA.

### A protein-coding ceRNA network in liver cirrhosis centers around SRC

**Fig 6:**
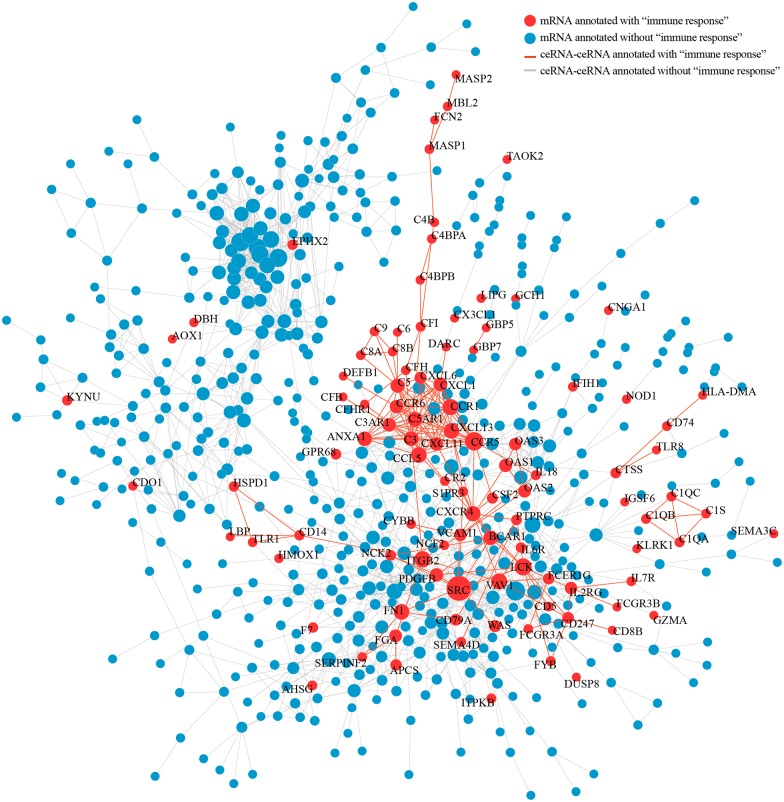
Protein-coding ceRNA interaction network in liver cirrhosis. The network was built using the String PPI database and the screened miRNA-mRNA pairs. Nodes represent ceRNAs. Node color indicates whether a ceRNA has at least one immune response related GO term (red for yes; gray for no). Node size is proportional to the degree of node itself. An edge indicates a miRNA linking corresponding ceRNAs. The SRC node had the highest degree out of the entire ceRNA—PPI network. The red genes were more concentrated and closely linked in the network.

We screened a total of 1137 ceRNA pairs in liver cirrhosis using the data from the screened miRNA—mRNA pairs in accordance with the theory that mRNAs binding to the same miRNA are regarded as ceRNAs. Next, we constructed the protein—protein interaction (PPI) network of protein-coding ceRNAs in liver cirrhosis using the results from the String PPI database. To identify ceRNAs related to the immune response in the network, we screened the genes related to the immune response according to the GO analysis results. Red nodes denote related genes that contained at least one GO term related to the immune response, whereas blue nodes denote unrelated genes. Edges denote two genes that are regulated by the same miRNA and can act as ceRNAs; red edges connecting two red nodes denote ceRNA pairs related to the immune response, whereas gray edges denote pairs that are not related to the immune response (Fig 6). The network was visualized through cytoscape software (S5 File). In the network, nine genes out of the top 20 nodes with the highest degrees were related to the immune response, including SRC (degree = 88), PIK3CG (degree = 25), GRB2 degree = 24), ITGAM degree = 22), CCR5 (degree = 21), STAT1 (degree = 20), PIK3CD (degree = 20), ETS1 (degree = 20), MEF2C (degree = 18). The SRC node had the highest degree out of the entire ceRNA—PPI network. Interestingly, the red genes were more concentrated and closely linked in the network. The red genes showed not only a wide range of interactions with blue genes but also showed extensive ceRNA interactions with other red genes. Multiple ceRNAs had crosstalk with SRC, such as SRC-ETS1-STAT1-CCR5-C3AR1, and SRC-PTPN1-PIK3CD-PI3KCG. Additionally, some paired red genes, such as CCR5-CCR6-C5-C5AR1, OAS2-MX1-XAF1, SRC-EST1-ITPR3, and SRC-PTPN1-PIK3CD formed a subnetwork.

### Overlapping DE GO terms in liver cirrhosis and HCC

**Fig 7:**
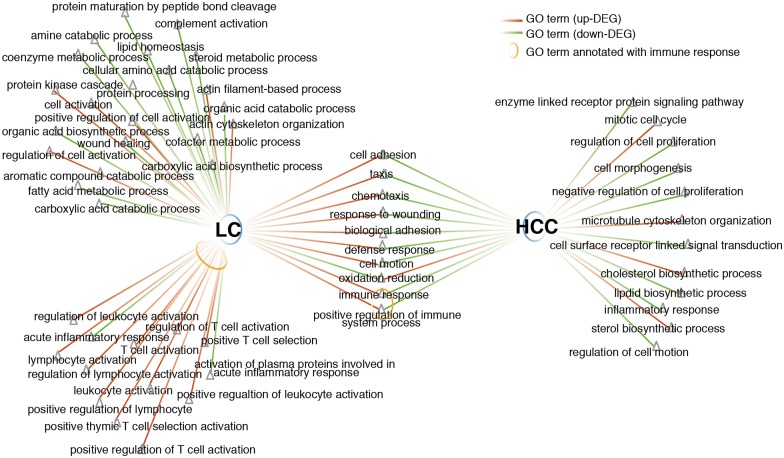
The overlapping GO terms in liver cirrhosis and HCC. Red edges, green edges and yellow circles represent upregulated GO terms, downregulated GO terms, and immune response-related GO terms, respectively. There are 10 overlapping DE GO terms in liver cirrhosis and HCC. “Immune response” was upregulated in LC, but downregulated in HCC.

We identified the DE mRNAs in HCC. We found 1449 DE mRNAs including 871 upregulated mRNAs and 578 downregulated mRNAs. GO analysis of DE mRNAs in liver cirrhosis and HCC was performed using the DAVID database. GO terms with a FDR<0.5 were considered as statistically significant. As shown in Fig 7, red edges represent upregulated GO terms, green edges represent downregulated GO terms and yellow circles represent immune response-related GO terms. There are 10 overlapping DE GO terms in both liver cirrhosis and HCC including “immune response”, “positive regulation of immune system process”, “cell adhesion”, “oxidation reduction” and so on. The GO term “immune response” was upregulated in liver cirrhosis, but downregulated in HCC. In contrast, the GO term “oxidation reduction” was downregulated in liver cirrhosis, but upregulated in HCC. Moreover, among the DE GO terms in liver cirrhosis, immune response-related GO terms occupied a very important proportion.

### Screening and GO analysis of overlapping DE genes in liver cirrhosis and HCC

**Fig 8:**
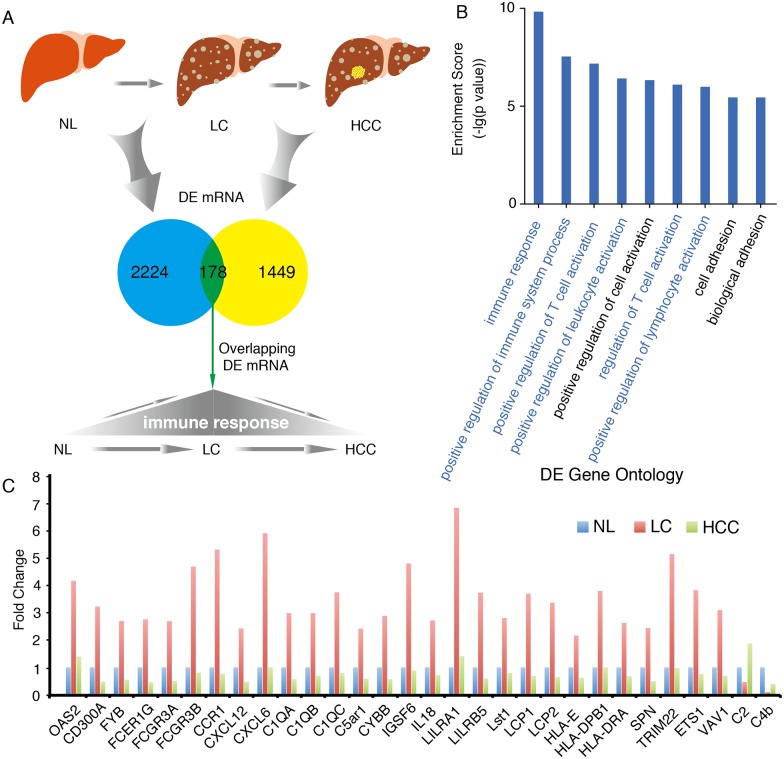
The overlapping genes and their GO terms in liver cirrhosis and HCC. (A) A total of 178 overlapping genes were identified in liver cirrhosis and HCC. (B) GO analysis of 178 overlapping genes. Six of the nine GO terms with a FDR <0.05 were related to the immune response. The GO term “immune response” showed the greatest statistical significance. (C) The expression of overlapping genes enriched in the GO term “immune response” in normal liver (NL), liver cirrhosis and HCC. The “immune response” GO term contained 30 genes, 28 of which showed markedly reduced expression in HCC as compared to liver cirrhosis and sometimes even lower expression than the NL group.

We screened overlapping DE genes in liver cirrhosis and HCC. A total of 178 overlapping DE genes were found in the two types of samples (Fig 8A). GO analysis was performed using DAVID. Six of the nine GO terms with a FDR <0.05 were related to the immune response. (S3 Table) The GO term “immune response” showed the greatest statistical significance (Fig 8B). This term contained 30 genes, 28 of which were downregulated in HCC but conversely upregulated in liver cirrhosis. Interestingly, the expression of CXCL6, CXCL12, and IL18 in the HCC group was even lower than in the normal group. C2 and C4b were slightly but not significantly upregulated in HCC (Fig 8C).

## Discussion

Clinically and pathologically, liver cirrhosis is a consequence of immune-mediated hepatocyte damage and repair processes. We used GO analysis of DE mRNAs in liver cirrhosis samples and found a large number of significantly different GO terms related to the immune response as compared to control samples. Our results confirm that liver cirrhosis and the immune response are closely associated. To further understand the pathogenesis of liver cirrhosis, we unveiled the regulatory coding and non-coding genes for the immunological processes that eventually lead to the development of liver cirrhosis.

Both TFs and miRNAs are essential for gene regulation in eukaryotes. The former regulates transcription and the latter regulates genes post-transcriptionally. The regulatory relationships between TFs and miRNAs are complex. There exists a triangular relationship of TF—miRNA—mRNA that can affect disease development and progression. For instance, HNF1α regulates the development and function of the liver through upregulation of miR-194 to suppress FZD6 expression[13]. In the present study, we identify a liver cirrhosis TF—miRNA—mRNA network containing 49 TFs, many of which have been previously implicated in liver disease (e.g. ETS1, FOS, FOXP3, FOXA2, and GATA2). Nishikawa et al. found that the expression of the TF FOXA2 was reduced in liver cirrhosis[14]. Cho et al. reported that overexpression of FOXA2 promotes the regeneration of liver tissues that have been damaged in liver disease, which suggests that FOXA2 is a negative regulator of liver cirrhosis[15]. The TF GATA2 can regulate PPARγ1 expression to activate HSCs[16]. Zhang et al. found that the TF E2F1 was a new profibrotic factor that could facilitate the progression of liver fibrosis through the Egr-1/SHP/EID1 network[17]. MYC promotes the progression of liver cirrhosis by upregulating the inflammatory factors IL8, IL10, and TGFβ[18]. All of these previous reports were validated in our TF—miRNA—mRNA network. Additionally, we found three important TFs (FOS, FOXP3, and ETS1) in the TF—miRNA—mRNA subnetwork of the immune response. FOXP3 is a well-known regulator of immune responses in T cells to promote the progression of liver cirrhosis. FOS activates natural killer (NK) and T cells to induce the development of liver fibrosis[19]. Our results suggest that these TFs can mediate liver cirrhosis-related gene expression through transcriptional regulation and interact with miRNAs to affect post-transcriptional gene regulation. Our results also uncovered several other TFs that may regulate the process of liver cirrhosis (e.g. FOXA1, RFX5, and ETS1) which are new potential targets for mechanistic studies of liver cirrhosis.

The results of our analysis on non-coding genes in the liver cirrhosis regulatory network validate the previous reports on 5 core miRNAs in this process (miR-203, miR-346, miR-590-5p, miR-219-5p, and miR-23c-5p). miR-203 is a regulatory factor for chronic inflammation and plays a role in chronic inflammation-mediated diseases[20]. miR-219-5p has been shown to regulate lymphoblastoid cells[21]. Song et al. found that overexpression of miR-203 suppressed TRPV4 expression in TGFβ-induced HSCs. TRPV4 promotes HSC proliferation, therefore the authors concluded that miR-203 is a liver cirrhosis inhibitor[22]. In the present study, we observed a significantly reduced expression of miR-203 in liver cirrhosis, which further supports Song’s conclusions. Qian et al. found reduced miR-346 expression in T cells from patients with cholestatic liver cirrhosis and suggested that miR-346 was involved in the immune response process[23]. miR-590 is expressed at low levels in patients with atrial fibrosis and renal fibrosis, resulting in reduced suppression of the target gene TGFβR2 and activation of the TGFβ pathway[24, 25]. The role of miR-590 in liver cirrhosis has not been described previously. Our analysis identified miRNA—target pairs in the gene regulatory network in liver cirrhosis (Fig 2). Therefore, miR-590-5p may also act as a regulatory factor for liver cirrhosis. Additionally, we found that miR-219-5p and miR-34c-5p were significantly downregulated in liver cirrhosis and played pivotal roles in the regulatory network.

To further understand the role of these miRNAs in liver cirrhosis, one approach is to uncover their target genes. For instance, the target gene of miR-219-5p is the TF FOXP3, which regulates T cell immunity to facilitate the progression of liver cirrhosis[26]. The target gene of miR-34c-5p is the important profibrotic factor NOTCH3, which activates HSCs to promote the development of liver cirrhosis [27]. Thus, miR-219-5p and miR-34c-5p have the potential to act as positive regulatory factors in liver cirrhosis.

Salmena et al. introduced the concept of ceRNA in 2011 which states that all types of RNA molecules (e.g. mRNA and lncRNA) can mutually regulate one another by competitively binding to miRNAs as long as they share common miRNA-binding sites[5]. In the present study, we evaluated the ceRNA effect in the liver cirrhosis 1ncRNA-miRNA-mRNA regulatory network and the immune response subnetwork. Our analysis of the liver cirrhosis lncRNA—miRNA—mRNA regulatory network revealed 11 lncRNAs (e.g. ENST00000575137, ENST00000592146, and ENST00000585445). None of the lncRNAs screened in the present study has previously been reported to act in the process of liver cirrhosis. As shown in our lncRNA—miRNA—mRNA network, the DE lncRNAs in liver cirrhosis mainly bind to miR-590-5p, miR-219-5p and miR-34c-5p. These three miRNAs regulate a considerable number of target genes. Therefore, some of the 11 lncRNAs we identified may competitively bind to the three miRNAs through a ceRNA effect and regulate the expression of their target genes. The immune response lncRNA—miRNA—mRNA regulatory network revealed seven DE lncRNAs. All of these DE lncRNAs were highly expressed with significantly high enrichment scores. Both the lncRNA—miRNA—mRNA and TF—miRNA—mRNA subnetworks were constructed using the same miRNA—mRNA pairs, and therefore the mRNAs in the two networks were identical. The DE mRNAs were enriched in GO terms related to the immune response in both subnetworks. Some of the DE mRNAs were shown to participate in the process of liver cirrhosis. In addition to FOXP3 mentioned above, CXCL11 has been reported to be significantly associated with liver cirrhosis following viral hepatitis, and overexpression of MEF2C can activate HSCs[28, 29]. These immune-related mRNAs, which are linked with liver cirrhosis, are target genes of miR-219-5p. Thus, the high expression of these mRNAs could be attributed to a ceRNA effect of lncRNAs that can also bind to miR-219-5p (i.e., ENST00000675137, ENST00000670663, and ENTS00000671336). Additionally, the target gene of miR-590-5p (CCR5) can facilitate the progression of liver fibrosis in mice, and the TGFβ receptor TGFBR3 plays a pivotal role in the development and progression of liver cirrhosis[30, 31]. The lncRNAs ENST00000692146, ENTS00000685445, and ENTS00000671336 may bind to miR-590-5p and reduce its inhibitory effect on these mRNAs. Therefore, the lncRNA—miRNA—mRNA subnetwork constructed in the present study uncovered important lncRNAs that might be involved in the immune response leading to liver cirrhosis, which provides new targets for mechanistic studies of liver cirrhosis.

In addition to the ceRNA effect of lncRNAs, mRNAs regulated by the same miRNA can also act as ceRNAs. We constructed the liver cirrhosis ceRNA—PPI regulatory network using the relationships described above and the PPI database (Fig 6). Interestingly, we detected ceRNAs related to the immune response in this network. The ceRNA—PPI network consisted of a total of 51 immune response genes; among which 40 genes constituted a subnetwork in liver cirrhosis. In addition to nodes linking two ceRNAs, complex regulatory relationships were also found: AXL can recruit GRB2; GRB2 can activate SRC; SRC can enable the phosphorylation of the NK cell regulator ETS1; and ETS1 can cause demethylation of FOXP3 resulting in more stable regulation of T cell functions[32–35]. The genes are connected in a series and form the ceRNA—PPI subnetwork of immune responses underlying liver cirrhosis. In our network, the tyrosine kinase SRC showed the highest node degree. Sabarinathan et al. found that the upregulation of SRC signaling promoted liver cirrhosis development and progression[36]. SRC regulates immune cell functions through phosphorylation. The liver cirrhosis ceRNA—PPI reveals a complex immune response regulatory network that contributes to the entire ceRNA—PPI network. The complex relationship includes non-coding genes that regulate coding genes and coding genes that mutually regulate one another. The complexity in the network illustrates the multidimensional mechanisms of immune gene regulation in liver cirrhosis. Our results provide new targets for mechanistic research and also potential targets for the treatment of liver cirrhosis.

To understand the role of the gene regulatory networok and immune response subnetwork in the progression of liver cirrhosis to HCC, we conducted overlapping GO analyses in liver cirrhosis and HCC. GO terms such as “immune response”, “cell adhesion”, and “oxidation reduction” in liver cirrhosis were inversely proportional in HCC. One possible reason for the reduction of in the term “cell adhesion” in HCC is that the expression of cell adhesion molecules is reduced in tumor cells after they are separated from inflammatory reactions and immune responses. This reduction in cell adhesion also correlates with the ability of cells in HCC to metastasize. “Oxidation reduction” was the only overlapping GO term that was upregulated in HCC. In liver cirrhosis, liver cell functions decline significantly. Pathogens such as hepatitis virus or alcohol destroy the oxidation-reduction reaction equilibrium in the liver and thus reduce liver cell function. When liver cirrhosis progresses into HCC, the changes in cell properties, the massive proliferation of tumor cells, and a substantial increase in energy metabolism lead to a significant increase in the oxidation-reduction reactions. Our results are consistent with the basic characteristics of the two disease processes. In the overlapping GO analysis, “immune response” was the GO term with the highest statistical significance. Interestingly, when we re-conducted the GO analysis on overlapping DE genes in liver cirrhosis and HCC (e.g., CCR1, CXCL6, and CXCL12), the results showed that these genes were mainly enriched in the same immune response terms (especially “T cell immune process”). T cells play a pivotal role in the process of tumor immunity. Soluble tumor antigen is processed by antigen-presenting cells after intake and then presented to helper T cells; the latter secrete cytokines to promote killer T cell proliferation and generate a specific killing effect. However, tumors can achieve immune escape through different pathways (e.g., antigen modulation of the tumor itself, induction of T cell apoptosis, and tumor-induced immunosuppression)[37, 38]. Among the overlapping DE genes of liver cirrhosis and HCC, 28 immune-related genes were downregulated during the progression of liver cirrhosis to HCC. This phenomenon may be a result of the immune escape of tumor cells. For instance, tumor cell antigen modulation allows the cells to escape from recognition by T cells and NK cells, thereby downregulating T cell- and NK cell-related immune factors (IL18 and CD300A)[39, 40]. Additionally, CCR1 is an important T cell immunity-mediating factor that regulates the immune response and tumor metastasis. Li et al. found that the immune activity of T cells was reduced after VAV1 downregulation[41]. This downregulation may be a mechanism for the immune escape of tumor cells. These immune-related overlapping DE genes in liver cirrhosis and HCC provide a new angle for understanding the immune escape mechanism of HCC and may lead to potential cancer treatment. The role of these immune factors in HCC following liver cirrhosis, either as a consequence or as a cause, warrants more in-depth analysis in the future.

In summary, we constructed liver cirrhosis TFs—miRNA—mRNA, lncRNA—miRNA—mRNA, and ceRNA—PPI regulatory networks and revealed the immune response subnetworks contributing to the constructed networks. We identified five miRNAs (miR-203, miR-219-5p, miR-34c-5p, miR-346, and miR-590-5p) that may play important roles in immune responses leading to liver cirrhosis. We also identified some important TFs, lncRNAs, and numerous ceRNA pairs implicated in immune responses leading to the development of liver cirrhosis. Finally, we conducted overlapping GO analyses in liver cirrhosis and HCC. GO terms such as “immune response”, “cell adhesion”, and “oxidation reduction” showed inversely proportional relationships in HCC as compared to liver cirrhosis. The coding and non-coding gene candidates identified in this macroscopic systematic study not only reveal new insights into the pathogenesis of liver cirrhosis but also provide many potential targets for the treatment of liver cirrhosis.

## Supporting information

S1 Table The differentially expressed coding and non-coding genes in liver cirrhosis. (PDF)

S2 Table The function of differentially expressed genes in liver cirrhosis. (PDF)

S3 Table The function of overlapping genes between liver cirrhosis and HCC. (PDF)

S1 File The Cytoscape file of the TF-miRNA-mRNA regulatory network in liver cirrhosis. (ZIP)

S2 File The Cytoscape file of the lncRNA-miRNA-mRNA regulatory network in liver cirrhosis. (ZIP)

S3 File The Cytoscape file of the TF-miRNA-mRNA regulatory network underlying the immune response in liver cirrhosis. (ZIP)

S4 File The Cytoscape file of the lncRNA-microRNA-mRNA regulatory network underlying the immune response in liver cirrhosis. (ZIP)

S5 File The Cytoscape file of the protein-coding ceRNA interaction network in liver cirrhosis. (ZIP)
