# Supplementary material for: Coding and non-coding gene regulatory networks underlie the immune response in liver cirrhosis
Source: PLoS One. 2017 Mar 29;12(3):e0174142. doi: 10.1371/journal.pone.0174142 (PMC5371304; doi:10.1371/journal.pone.0174142)
Supplement: S1 Table — (PDF) [file pone.0174142.s001.pdf]

Supplementary Table 1. The differentially expressed coding and non-coding genes in liver cirrhosis.

lncRNA Symbol	log2FC	P.Value	FDR
SMIM12-001	-2.428026518	2.52E-07	0.000903833
FAM99B-001	-3.104556194	0.000102662	0.028274226
RP11-535A19.1-001	-2.234307887	6.48E-06	0.007736147
PLEKHG6-001	-1.239219352	0.00036209	0.049953853
PLEKHG6-003	-1.239219352	0.00036209	0.049953853
TUSC8-001	-2.192614128	7.89E-05	0.024569693
LINC00238-006	-1.463838638	6.49E-05	0.024092451
LINC00238-001	-1.463838638	6.49E-05	0.024092451
LINC00238-005	-1.463838638	6.49E-05	0.024092451
LINC00238-004	-1.463838638	6.49E-05	0.024092451
LINC00238-002	-1.463838638	6.49E-05	0.024092451
ABAT-004	-1.450677846	3.55E-05	0.021772174
U91319.1-002	-2.30367014	1.54E-05	0.016574622
ABCC6P1-004	-3.352301892	4.78E-05	0.023328775
AOC4P-006	-2.565863472	0.000208042	0.042232157
HPN-AS1-001	-2.296245175	0.000302842	0.045674063
CYP2B7P-006	-3.651062474	0.000231955	0.042955756
CYP2B7P-001	-3.651062474	0.000231955	0.042955756
DGCR5-001	-2.373700203	0.000100774	0.028274226
C1QTNF6-002	-1.630286065	4.68E-05	0.023328775
RP11-71E19.1-001	-1.638610195	0.000197158	0.042232157
RP11-372E1.4-001	-3.133938887	6.71E-05	0.024092451
RP11-328K4.1-002	-2.79053328	7.05E-05	0.024092451
LINC01554-003	-4.384885728	1.58E-08	0.000170111
LINC01554-001	-3.407856568	8.76E-07	0.002351978
AC004538.3-010	-2.557557759	3.77E-06	0.006744772
AC004538.3-001	-2.439392813	5.57E-06	0.007475873
AC004538.3-004	-2.557557759	3.77E-06	0.006744772
CTA-315H11.2-001	-2.537697365	0.000252307	0.043209056
RP11-115J16.1-001	-3.118141739	4.10E-08	0.000220044
ATP8B5P-001	-1.187034919	0.000217806	0.04253553
PCAT7-001	-1.151206751	0.000250438	0.043209056
SNHG12-002	1.119232528	0.0002588	0.043209056
ENAH-004	1.518121133	0.000324978	0.047816254
MARVELD1-001	2.047279763	2.41E-05	0.021554673
RP11-693N9.2-002	1.445113959	8.01E-05	0.024569693
RP11-693N9.2-009	1.445113959	8.01E-05	0.024569693
RP11-617F23.1-001	1.498338759	0.000306166	0.045674063
MCTP2-004	2.425649733	0.000130288	0.034132304
CTD-2135D7.2-002	1.530047056	0.000334616	0.047921487
CTD-2135D7.2-001	1.530047056	0.000334616	0.047921487

CTD-3195I5.1-001	1.270080848	0.000208095	0.042232157
CTD-2020K17.1-001	1.705608679	0.000251817	0.043209056
C1QTNF1-AS1-001	2.987848786	0.000291141	0.044673501
C1QTNF1-AS1-002	2.987848786	0.000291141	0.044673501
CTC-241F20.3-001	2.139399464	0.00010707	0.028750984
ZNF667-AS1-003	1.551308409	4.05E-05	0.021772174
ZNF667-AS1-001	1.551308409	4.05E-05	0.021772174
ZNF667-AS1-004	1.551308409	4.05E-05	0.021772174
ZNF667-AS1-008	1.551308409	4.05E-05	0.021772174
ZNF667-AS1-002	1.551308409	4.05E-05	0.021772174
ZNF667-AS1-005	1.551308409	4.05E-05	0.021772174
ZNF667-AS1-009	1.551308409	4.05E-05	0.021772174
RNF144A-AS1-005	1.98558292	0.000242559	0.043209056
RNF103-CHMP3-002	1.074602891	0.000284082	0.044673501
ANKRD36BP2-003	1.410132644	0.000261483	0.043209056
ANKRD36BP2-001	1.410132644	0.000261483	0.043209056
MBD5-001	1.491917021	0.00036276	0.049953853
LINC00471-001	1.261300425	0.000186286	0.042232157
C22orf34-002	2.039295427	0.000226455	0.042955756
DCP2-004	1.650563039	0.000201334	0.042232157
BTN3A2-017	2.013105118	6.38E-05	0.024092451
FABP5P3-001	2.466494317	5.40E-06	0.007475873
PGM5P3-AS1-001	1.990267691	7.18E-05	0.024092451
PGM5P3-AS1-003	1.990267691	7.18E-05	0.024092451
DOCK8-018	2.196289158	8.64E-05	0.025093977
DOCK8-001	2.356207626	1.82E-05	0.017767296
DOCK8-010	2.196289158	8.64E-05	0.025093977
GGTA1P-005	1.960989542	0.000276707	0.044359825
GGTA1P-004	1.960989542	0.000276707	0.044359825
mRNA Symbol	log2FC	P.Value	FDR
NAT2	-2.087901987	0.004749354	0.042139419
AGT	-3.278357195	0.000251855	0.01169459
AGXT	-2.45738476	0.000736365	0.016396702
ALDOB	-3.272684994	0.000578076	0.014895881
APOA1	-2.257465921	0.001304911	0.020787074
APOC3	-1.995393923	0.001634386	0.023448789
APOH	-2.672517655	0.000564534	0.014719698
AR	-2.625980991	0.000759156	0.016486247
ARG1	-3.947734534	0.001246025	0.020340824
ARSE	-2.762312508	0.00068395	0.015932657
BCKDHB	-2.090016193	0.002814545	0.031010118
C2	-1.099629192	0.001345805	0.021135344
C3	-2.062344485	0.000477275	0.013647112
C6	-3.039797035	0.001727788	0.024144095

C8B	-3.590886114	0.001153355	0.019497347
CA2	-2.054082636	0.000296321	0.012102504
CBS	-2.900110055	0.000425208	0.013045227
CNGA1	-2.317184994	0.001416004	0.021775325
EPHX1	-1.551141727	0.000923984	0.017880184
F11	-3.649967542	0.000277472	0.012003271
F5	-2.534308686	0.000330411	0.012254849
F7	-1.831650871	0.003530313	0.035364733
F9	-2.993700966	0.000449199	0.013349109
FANCC	-1.694492129	0.000878765	0.017658338
FAH	-2.038676961	0.001697548	0.023884852
FGFR3	-2.983527857	0.000391796	0.012854122
FH	-1.267109278	0.002461116	0.028868277
G6PC	-2.868192584	0.001547396	0.022793387
GALK1	-2.096319242	8.83E-06	0.004370993
GAMT	-2.183969519	0.001418553	0.021775325
GCDH	-1.902655763	0.001088879	0.019033012
GCH1	-1.171612154	0.004295732	0.039893195
GHR	-2.327712671	6.36E-05	0.008063947
GJB1	-2.974251359	0.003090838	0.032862276
GLDC	-2.700904506	0.000651856	0.015472375
SERPIND1	-2.427979322	0.000668989	0.015720572
CFH	-2.339480292	0.001544947	0.022773745
HGD	-3.132783082	0.002545307	0.029375342
HMGCL	-1.541181925	0.004001961	0.038399232
CFI	-1.887685423	0.00057732	0.014895881
LCAT	-2.065892481	5.93E-05	0.008053145
LIPC	-3.448604587	0.000438994	0.013160785
MAOA	-2.044386618	0.001594838	0.023141585
MBL2	-2.816473431	0.002309532	0.027873334
MET	-1.344411857	0.004297863	0.039893195
MTTP	-3.155994198	0.002199513	0.02724349
MUT	-2.206485326	0.001121787	0.019278478
PAH	-3.027400324	0.001640913	0.023472434
PCBD1	-1.15630032	0.003372812	0.034395268
PKLR	-1.284312276	0.001615235	0.023255735
PLG	-2.660918903	0.000794725	0.016744182
PROC	-2.700883871	0.00020285	0.011049475
PROS1	-1.428259878	0.001334396	0.021029389
PTH1R	-1.358934467	0.000101747	0.008967806
SLC2A2	-3.014495818	0.000516444	0.014145023
SLC3A1	-1.547133006	0.002269998	0.027705534
SRD5A2	-3.473763322	0.000301744	0.012141407
TAT	-3.115324786	0.001771048	0.024504666

SERPINA7	-3.223595853	0.002939368	0.03185251
TTPA	-2.731952625	0.000893279	0.017740455
TTR	-3.790152325	5.41E-05	0.007939072
XDH	-2.810807836	0.001095463	0.019082733
APOB	-2.616351879	0.000723952	0.016277754
ABCC2	-2.98788657	0.001933874	0.025605119
MAT1A	-3.306927184	0.000801182	0.016828126
PON1	-3.610309381	0.000390161	0.012854122
HNF4A	-3.505801858	0.000405544	0.012957099
HNF1B	-2.580179554	0.004675051	0.041862213
ALB	-2.85899328	0.00031079	0.012234335
AMT	-1.065955709	0.002425038	0.028599867
APOC2	-2.799085906	0.001834246	0.024948682
SERPINC1	-2.335651926	0.000719763	0.016240629
F10	-2.359849305	0.001062271	0.018907867
FGA	-3.264645374	0.001305301	0.020787074
FGG	-2.058964722	0.001808689	0.024732886
OTC	-2.682852901	0.002373037	0.028233055
PCCB	-1.712448732	0.002249249	0.027531776
C8A	-3.023230573	0.00223204	0.027430074
IL6R	-1.614555793	0.003414316	0.034573951
GC	-2.128266692	0.000866771	0.017493386
CD14	-1.276748919	0.001902924	0.025399147
IGFBP1	-3.13644695	0.003873228	0.037629817
ORM1	-2.332961077	0.002073518	0.026507793
ORM2	-1.489914384	0.000695741	0.016028594
SERPINA5	-1.548317365	0.000739589	0.016396702
VTN	-2.106368369	0.000998262	0.018471549
IL13RA2	-1.068472452	0.00240288	0.028406019
AGL	-1.348641674	0.002115661	0.026828519
AGL	-1.465216267	0.000247982	0.01169459
ABAT	-3.26292504	0.000555905	0.014663262
ADH1A	-1.900200735	0.000617677	0.015131243
ADH1B	-2.650867205	0.000637713	0.015335149
ADH1C	-1.59089665	0.003584405	0.035645009
ADH4	-2.530469011	0.001322488	0.020978947
ADH6	-2.852435738	0.000217337	0.011351772
ALAS1	-2.021101435	0.00253553	0.029295628
ALDH1A1	-1.480621924	0.001442594	0.021975426
ALDH2	-1.600745059	0.002150634	0.027086582
ALDH1B1	-1.740898456	0.000655712	0.015512186
ALDH9A1	-1.131941072	0.001121537	0.019278478
C4BPA	-3.144961743	0.002695931	0.030242329
C4BPB	-3.376583628	0.001035052	0.018646769

CYP2A6	-3.888251351	0.00055363	0.014663262
CYP2A7	-3.757368372	0.000475049	0.013629768
CYP3A7	-2.721500342	0.002112167	0.026828519
CYP2A13	-3.399716411	0.002170978	0.027171364
CYP2C19	-2.951007891	0.000583555	0.014912856
CYP2C8	-2.715627437	0.0015636	0.022899894
CYP2C9	-3.1601974	0.001242937	0.020340824
CYP2C18	-2.595609351	0.000818793	0.017057636
CYP2E1	-2.162174775	0.000327578	0.012254849
CYP2J2	-2.535463165	0.005911001	0.048407141
CYP3A5	-3.958382518	0.000563727	0.014717464
CYP4A11	-3.140938553	0.000368633	0.012603013
CYP7A1	-2.702356757	0.002805295	0.030952293
CYP51A1	-1.958782244	0.000988604	0.018449123
DBH	-2.724731166	2.91E-05	0.006533081
DDC	-3.090190979	0.001190832	0.019879011
DRD1	-1.741139888	0.000691052	0.016014214
GGCX	-2.419911827	0.001367628	0.021379402
GSTA2	-3.468072531	3.42E-05	0.007011546
KNG1	-2.471433945	0.001929691	0.025598816
CYP4F3	-2.998015824	0.000517533	0.014145023
MAOB	-2.231648232	0.001791428	0.02460588
MME	-2.109731584	0.00020144	0.011049475
NQO2	-1.446313594	0.000694267	0.016028594
PC	-2.794449992	0.001894409	0.025361483
SERPINF2	-2.97749369	0.00060224	0.015063312
PON3	-3.032458634	0.000924973	0.017880184
DNAJB3	-2.906480676	4.53E-05	0.007487582
UGP2	-1.41291048	0.001295817	0.02073307
RORC	-2.243692627	0.00043294	0.013136157
TM6SF2	-1.783370589	0.000709197	0.016141555
IL17REL	-1.387878626	0.003432996	0.034683141
OR13C3	-1.235791783	0.000199092	0.011049198
C4B	-1.078909656	0.003109151	0.032943118
FMO3	-3.276377407	0.000660259	0.015583632
FCRLB	-1.459585757	0.002055588	0.02642751
TPD52L1	-1.512493646	0.002238589	0.027460704
TPD52L1	-1.951822471	0.001126338	0.019278478
AGMO	-1.933544093	0.000622738	0.015171623
OR4D10	-1.650813323	0.004947313	0.043197604
AK4	-1.528403592	0.000757013	0.016486247
HAO2	-4.007949348	0.000171587	0.010584994
ERBB3	-1.596838709	0.000669141	0.015720572
SDC1	-2.534877352	0.001278432	0.020587681

SCP2	-1.32679455	0.001891731	0.025351442
AKR1CL1	-2.258267605	0.000495443	0.013990274
GALE	-1.194089811	0.000337768	0.012254849
TCP1	-1.741678583	0.002008986	0.026170303
SLCO1B7	-2.599059991	0.003568704	0.035575517
ACSM2A	-3.824937615	0.000339529	0.012254849
C10orf114	-2.406452336	0.000622201	0.015171623
CYP4A22	-3.209683762	0.000278464	0.012003271
AFMID	-1.80223949	0.001765794	0.024474169
AR	-2.302979192	0.001034426	0.018646769
PLIN5	-2.793212609	0.001079983	0.01901645
STAP2	-1.886407186	0.001890343	0.025351442
CFH	-2.122540929	0.001372672	0.021441826
FOLH1	-2.934073933	9.75E-05	0.008856782
PAPSS2	-1.401153859	0.002767273	0.030688023
DNAJC25	-1.70135901	0.002597796	0.029612849
ARID3C	-1.515525358	0.002286074	0.027739133
C4BPB	-2.693542569	0.001821644	0.024843478
MSMO1	-2.168143359	0.000311327	0.012234335
ZBTB16	-2.031486835	1.23E-05	0.004370993
PCK2	-2.530783108	0.000350918	0.012360149
GCH1	-1.25547639	0.001455592	0.022127597
GPHN	-1.479395092	0.005293218	0.04521604
ASPDH	-2.348602895	0.000215434	0.011335849
ASL	-1.520708031	0.001475235	0.022309928
SC5DL	-1.801574519	0.000533275	0.014363257
CES1	-1.522723045	0.004900716	0.042941209
ANKRD56	-2.736905771	0.000264633	0.011874189
ALDH3A2	-1.208575783	0.004693004	0.041868904
LONRF3	-1.407855497	0.005001739	0.04354606
SERPING1	-1.57467584	0.002469108	0.028904426
PBLD	-2.040039168	0.004342599	0.040122117
SAR1B	-1.121662642	0.005498208	0.046396503
FAM134B	-1.380503857	0.000958915	0.018127049
SDHC	-1.181162404	0.001784736	0.024603081
PALM2	-1.697290906	0.001133748	0.019288835
PHYH	-2.10918899	0.000688426	0.015989647
HSD17B10	-1.262741108	0.001606822	0.023200054
TTPAL	-1.703547869	5.40E-07	0.001836216
GPER	-1.416119952	0.000106658	0.009156966
PC	-2.829615415	0.001585356	0.02305315
NUPR1	-1.009955747	0.001883719	0.025334653
NET1	-1.610494124	0.002253337	0.027563891
TUBB2A	-1.828432805	0.005090683	0.044057391

UGT2B11	-2.302572672	0.003350957	0.034238845
UGT2B7	-2.431116642	0.000384299	0.012777971
UGT2B10	-2.6395693	0.000417562	0.013005203
UGT2B15	-3.383685544	0.000605344	0.015107025
CDC14B	-1.42671472	0.003995353	0.038399232
MYH14	-1.429599819	0.005366804	0.045634597
PDE11A	-1.275739866	0.002575607	0.029524757
DEPDC7	-2.934716659	0.001641783	0.023472434
NR1I3	-3.315344482	0.000415899	0.013005203
NR1I3	-3.4071619	0.000390707	0.012854122
NR1I3	-3.473146545	0.00028896	0.012095493
NR1I3	-1.678017878	0.001984287	0.026002052
NR1I3	-3.245742008	0.000985788	0.018413405
NR1I3	-3.508182119	0.000646625	0.015431998
NR1I3	-3.476931523	0.00063393	0.015298251
NR1I3	-3.324643174	0.000739205	0.016396702
EXOC3L4	-1.470009171	0.000142612	0.009971142
RGPD2	-2.149585725	0.003057432	0.032646723
SLC29A1	-1.249002838	0.00017676	0.010584994
SLC29A1	-1.417423461	0.001053181	0.018828145
PAICS	-1.024249901	0.003601969	0.035740345
DBI	-1.202207835	0.000791468	0.016692777
ALDH5A1	-2.174785323	0.000211467	0.011243021
TTC36	-1.488182933	0.002851817	0.03125108
SERPINA11	-3.103617339	0.004394183	0.040372727
ASPG	-1.24577963	0.000229702	0.011445469
ONECUT3	-1.880541951	0.001543836	0.022773745
CPN2	-3.559294259	0.000600082	0.015063312
CCDC150	-1.824176377	0.003820375	0.037252611
CYP4F2	-3.647411612	0.000292338	0.012095493
SCARB1	-1.435432649	0.003194748	0.033454343
ZGPAT	-1.742120299	0.000475333	0.013629768
DDT	-1.657246925	0.003136	0.033124526
C2orf54	-2.256148519	0.002203984	0.027254108
AADAC	-3.490924064	0.00141671	0.021775325
ANG	-1.902109131	0.002470584	0.028905126
GJB1	-2.97708483	0.000983074	0.018401912
HMGCS1	-1.684500778	6.94E-05	0.008226635
C10orf125	-1.346392418	0.000366671	0.01259222
SLC17A3	-2.713092648	0.003151833	0.033220352
COQ10A	-1.002276748	0.003823866	0.037263982
CLEC1B	-2.028616788	0.000878758	0.017658338
NPW	-1.662779869	0.002186853	0.027219455
HOOK2	-1.174986812	0.000245888	0.01169459

TMEM192	-1.075049237	0.001479962	0.022344068
SERPINA10	-2.148809932	0.001045424	0.018754179
10-Mar	-1.037828052	0.006201824	0.049868622
NPC1L1	-3.123668937	4.06E-05	0.007183485
KNG1	-3.21649399	0.000666616	0.015697374
ADH6	-3.402846071	0.000163901	0.010424332
C4orf19	-1.952355215	0.000778226	0.016576452
FGGY	-1.864501825	0.004094886	0.038794056
ASPDH	-1.94893058	0.000335267	0.012254849
C6	-3.681676592	0.000414167	0.013005203
CPS1	-2.699550458	0.001511667	0.022576581
CPS1	-2.066823468	0.003412647	0.034573951
ADK	-1.889393611	4.46E-05	0.007487582
FOXD4L5	-1.052863995	0.001124934	0.019278478
ODZ2	-1.044440627	0.00511717	0.044230376
RGPD6	-1.732091324	0.006127007	0.049481396
ABAT	-2.958695763	8.70E-05	0.00872223
BAAT	-2.652712688	0.000768769	0.016486247
GALE	-1.036297933	9.28E-05	0.008734636
SLC6A12	-2.78630168	0.000484709	0.013790312
PRG4	-2.985525507	0.000676671	0.015860984
CLCN5	-1.257964942	0.001682339	0.023755519
SLC6A12	-2.016941002	8.47E-05	0.00872223
CYP2C18	-3.199020948	0.001079452	0.01901645
CYP4F11	-1.714425774	0.005599363	0.046774385
GBA3	-2.782819541	0.000218725	0.011351772
CRYZ	-1.221536667	0.002339789	0.028033532
MYO1B	-1.889822256	0.00492481	0.043059922
SLC39A14	-2.258974863	0.000999377	0.018471549
GNE	-2.40676967	0.000146084	0.010072696
HOGA1	-2.68601872	0.002534249	0.029295628
SARDH	-3.094788092	0.000343962	0.012360149
SIGIRR	-1.221447297	0.001328248	0.02100504
SIGIRR	-1.11129534	0.0010101	0.018505755
SH3D19	-1.24790756	0.003407153	0.034565756
CRYZ	-1.620177195	0.0054144	0.045962745
SLC39A8	-1.002302757	0.001640782	0.023472434
SLC39A14	-2.46140955	0.001392797	0.021623837
ELFN1	-2.423263833	4.31E-05	0.007335659
SLC39A5	-3.069733406	0.000151374	0.01013002
IGSF9	-1.37507789	0.000422404	0.013045227
ACBD4	-1.286309283	0.001039465	0.018681087
AFM	-2.888159953	0.000316383	0.012254849
POSTN	-2.525082791	2.79E-05	0.006427112

DHRS1	-1.529553615	0.000120741	0.009554452
HSD17B13	-3.63155827	0.000535506	0.0143951
LRP5L	-1.001089844	0.001532803	0.022715011
AKR1C2	-2.349801897	0.005807068	0.047869821
GPT2	-2.601898559	0.002242402	0.027479514
SLC38A4	-3.064858067	0.000473747	0.013629768
POSTN	-1.574750711	0.002115689	0.026828519
UNC93A	-1.808088531	0.004212137	0.039431204
CES5A	-2.481655369	0.000170866	0.010584994
NHSL1	-1.733054857	0.001136035	0.019311653
ANPEP	-2.811258404	0.000175388	0.010584994
AOX1	-3.250077238	0.000506135	0.014054629
AZGP1	-2.318799049	0.00274767	0.030520361
GGCX	-2.270665999	0.000990595	0.018468674
TMEM25	-1.240788559	0.002573706	0.029521318
FMO5	-3.352795826	0.000808475	0.01694644
ANG	-2.671519489	0.001278951	0.020587681
PRAP1	-2.759371014	0.001023116	0.018570027
CLDN3	-1.033447608	0.004464431	0.040826872
CPN1	-2.390236722	0.003290154	0.033942283
HSD3B7	-1.15357396	0.000646805	0.015431998
FMO5	-2.644462487	0.001555542	0.022822466
ALKBH2	-1.090746002	0.005855995	0.048111064
C2orf72	-2.975612198	0.001009071	0.018505755
PALM3	-2.119873519	0.000525602	0.014288541
SLC13A5	-2.560598627	0.000258353	0.011721179
RASSF7	-1.305720664	0.004179801	0.039304317
PLEKHG6	-1.203671658	0.004573551	0.041391236
FGFR2	-2.332171666	0.003578436	0.035645009
OGDHL	-2.922851662	0.001555453	0.022822466
SCG5	-1.977252474	0.000781081	0.016576452
UGT2B10	-3.431691322	8.89E-05	0.00872223
FGFR2	-2.013029765	0.004377668	0.040240641
ENTPD5	-2.437964535	0.00208578	0.026597929
AFMID	-2.347785295	0.00101158	0.018505755
LSS	-1.53636055	0.003346536	0.034238845
AKR1C1	-2.615212699	0.001654937	0.023610904
DDT	-1.890423845	0.002710048	0.030291976
DECR1	-1.208796666	0.003624397	0.03583195
FABP1	-2.026467086	0.00589395	0.048306259
FMO5	-2.898270955	0.001608498	0.023207834
ASGR2	-2.658588538	0.002070338	0.026507793
GATM	-2.068864912	0.000366287	0.01259222
CXADR	-1.430858535	0.004480849	0.040876238

AKR1C2	-2.915083822	0.002701901	0.030242329
HGFAC	-2.276376117	0.003312822	0.034072833
DPYS	-2.902580129	0.000297301	0.012102504
GPLD1	-3.208692164	0.000204013	0.011049475
GSTZ1	-1.330044377	0.001037671	0.018665282
ECM2	-2.255453024	0.000127069	0.009595296
ACADL	-2.517502561	0.000284351	0.012019591
AHSG	-1.958735617	0.003281882	0.033897042
ALCAM	-1.826391334	0.003591425	0.035662712
APOF	-3.032673867	0.003226174	0.033645663
CPS1	-2.718178117	0.000967362	0.01823606
ARSD	-1.136845284	0.004962947	0.043263672
APCS	-2.768598159	0.000612096	0.015131243
ASGR1	-2.813011153	0.002287668	0.027739133
BHMT	-3.472656971	0.000337722	0.012254849
ACADSB	-2.700738214	0.000290747	0.012095493
AMBP	-1.16598157	0.003626019	0.03583195
MASP1	-2.319590045	0.004006189	0.038399232
APOC4	-2.422652712	0.000373726	0.012603013
C9	-2.924626271	0.004162022	0.039193655
EGR1	-1.758416264	0.001989989	0.026043337
EPHX2	-2.418975286	0.00139744	0.021636005
C5	-3.018928107	0.000844322	0.017306885
CDO1	-2.052485204	0.001126013	0.019278478
DSG1	-1.753637895	0.002072978	0.026507793
FN1	-1.184594634	0.002885541	0.031537042
GOT2	-1.81057616	0.000257392	0.011720495
HPD	-2.230365549	0.001708447	0.023922942
BAAT	-3.012103578	0.001141013	0.019364032
CYB5A	-1.490162438	0.001161122	0.019546423
EHHADH	-2.671122368	0.001006584	0.018505755
ACSL1	-1.817583556	0.002365553	0.028176851
FMO4	-1.259597181	0.002205521	0.027256614
AKR1C4	-2.995886108	0.000818175	0.017057636
CFB	-1.55727827	0.000945486	0.018068448
CA5A	-2.261821865	0.003517457	0.035306003
CRYM	-1.811153215	0.005647859	0.047073987
ECI1	-1.327088485	0.002353866	0.028136142
CRYZ	-1.871238328	0.002397906	0.02836989
CTPS	-1.664243345	0.000890427	0.01771828
CFHR1	-2.548630201	0.001278496	0.020587681
CPB2	-3.29056507	0.000418116	0.013005203
ENO3	-2.559244886	2.37E-05	0.006193459
GATA4	-2.307189385	0.001385296	0.021556556

IVD	-1.1744521	0.00311111	0.032944201
ETFB	-1.675744724	0.000471353	0.013629768
HSD17B2	-2.817206827	0.003470168	0.034934394
INHBA	-1.678396297	0.000934366	0.01793028
LECT2	-3.072119945	0.000149631	0.010125656
ID1	-1.732413247	0.000243002	0.011672752
HMGCS1	-1.708477186	0.001210465	0.020026719
HLF	-1.223972278	0.006169061	0.049742316
OCLN	-2.280964314	0.000844959	0.017306885
ITIH4	-2.709261023	0.001435752	0.021911473
KCNJ6	-1.249277752	9.48E-05	0.008797851
HSPE1	-1.048969904	0.002480969	0.028993393
NTHL1	-1.57096468	0.00298866	0.032230901
ITGA5	-1.278836228	0.00088709	0.01771828
PLGLB2	-2.256336338	0.002761501	0.03064066
ITIH2	-2.828787391	0.000743457	0.016426854
PER1	-1.394945182	0.001420414	0.021775935
PPL	-1.987897229	2.44E-05	0.006193459
FURIN	-1.492209049	0.000855371	0.017351477
PCK1	-1.440034704	0.002314473	0.027876304
SERPINF1	-2.108878251	0.000170776	0.010584994
PGM1	-1.968641061	0.000954096	0.018086189
PRG2	-1.70083006	0.005540391	0.046571575
PFKFB1	-2.355448419	0.000231166	0.011475579
PLS1	-2.357183122	0.00182506	0.024857388
PROX1	-1.079000066	0.006106425	0.049412907
PTPRF	-1.976924271	0.002550732	0.02942133
PCYT2	-1.988345297	0.00114696	0.019448002
PRPSAP1	-1.278920576	0.000975404	0.018320015
MAPK13	-1.039934722	0.001332831	0.021029389
PEBP1	-1.110062451	0.001485205	0.022362968
PCDH1	-2.066391762	0.005978589	0.04878452
RDH5	-1.253697237	0.005581463	0.046723725
SDC1	-2.447862455	0.002172006	0.027171364
SDC4	-1.494677832	0.00078767	0.016664319
PTPRD	-1.418723868	0.001795437	0.02460588
PLIN1	-2.716914647	5.01E-05	0.007805712
SLC22A1	-2.783450668	0.000222971	0.011408959
TFR2	-1.990843458	0.001200987	0.020015789
CX3CL1	-1.056481171	0.001534921	0.022715011
RARRES2	-1.342362733	0.003531853	0.035364733
SLC6A1	-2.771309297	0.000911289	0.017877478
SCP2	-1.584072432	0.00167827	0.023744664
SORD	-1.980283767	0.000137598	0.00984617

SLC6A12	-2.557903845	0.000412473	0.013005203
TM7SF2	-1.682390626	0.000780558	0.016576452
UGDH	-1.719733795	0.000721721	0.016245481
SLPI	-1.290633143	0.002569057	0.029499358
TCEA3	-2.113423379	0.000765952	0.016486247
TPSAB1	-1.077214808	0.002091507	0.026654314
TST	-1.897717472	0.001661346	0.023669247
THOP1	-1.6071498	0.004873869	0.042890043
UAP1	-1.787080513	0.001091625	0.019064259
SQLE	-1.247442215	0.001024189	0.018570027
VSNL1	-1.895714765	0.000856695	0.017351477
TSPAN6	-1.482923247	0.001268739	0.020501658
ACOX2	-1.724639937	0.000589439	0.014967647
SLC10A1	-3.080763335	0.001247528	0.020340824
THRSP	-3.910753624	1.04E-05	0.004370993
SPR	-1.733018226	0.002125094	0.02692428
TPD52L1	-1.946657907	0.001402175	0.021670549
FCN3	-1.616644276	0.003907981	0.037847012
WEE1	-1.339649044	0.001896126	0.025366557
HSD17B6	-1.206473946	0.00367416	0.036080643
NIPSNAP1	-1.636919182	0.000602134	0.015063312
ANXA9	-1.434762831	0.000310667	0.012234335
ZKSCAN1	-1.319856918	0.000324097	0.012254849
ALDH4A1	-2.555659078	0.000111053	0.009156966
KMO	-1.509945823	0.002007178	0.026167651
GPAA1	-1.119080731	0.002795401	0.030880359
GGH	-2.357132329	0.001242704	0.020340824
AKR1C3	-3.173764228	0.001046288	0.018754179
NR5A2	-3.012125274	0.002691158	0.03023813
ABCC3	-1.487860949	0.005567016	0.0466569
PEX11A	-1.700488461	0.000997864	0.018471549
KCNK5	-1.079569408	0.004846082	0.042719177
CHRD	-1.99544268	0.000953894	0.018086189
NR1I2	-3.478301483	0.000183011	0.010584994
SELENBP1	-1.363774635	0.000292516	0.012095493
SLC27A2	-3.373509788	0.002138592	0.026995504
KYNU	-1.722014527	0.00456121	0.041313962
ABCB11	-2.554812739	0.005730013	0.04751582
GYG2	-2.680474956	0.000333738	0.012254849
ACOX1	-2.414596837	0.001100829	0.019128638
SLC1A2	-2.811416273	0.004979952	0.043393388
GJB2	-3.137759817	0.001084053	0.01901645
GDA	-2.4958229	0.000789927	0.016692777
STBD1	-2.595543389	0.003564163	0.035547611

ECHS1	-1.921329487	0.00014135	0.009971142
SYT7	-2.610553221	0.000124531	0.009595296
ACVR1B	-1.022970664	0.005107582	0.044166199
SHMT1	-1.511669037	0.001921276	0.025536956
SLC28A1	-2.374921636	4.16E-07	0.001836216
CTNNA2	-1.317864987	0.000809639	0.016951958
BBOX1	-3.543488696	3.15E-06	0.00313707
HABP2	-3.28304873	0.000395799	0.012854122
FASN	-1.888218128	0.000320906	0.012254849
FADS2	-2.057904545	0.000255196	0.01169459
FCN2	-1.534807761	0.002313564	0.027876304
LBP	-3.11557343	0.004363564	0.040177712
HSD17B10	-1.168701203	0.0046132	0.041549015
ONECUT1	-2.349184713	0.002154761	0.027112966
CEBPA	-1.591396575	0.000528139	0.014292776
RCAN1	-1.592373073	0.002044989	0.026341005
DSP	-1.59002851	0.005523981	0.046487057
PCK2	-2.021903064	0.001093468	0.019064259
MTMR4	-1.526679302	0.000451284	0.013391597
REPS2	-1.007076062	0.004410256	0.040430979
PKP2	-2.189005212	0.001942908	0.025665926
FOXA3	-2.156493933	0.000131562	0.00971712
LRAT	-1.600882081	0.002909699	0.031648593
FOXA1	-3.100892545	0.000706139	0.016099625
NTN1	-1.716726689	0.000227525	0.011445469
CDH1	-1.605201112	0.004136026	0.039033531
CYP8B1	-2.842933685	0.00324407	0.033774058
RGN	-1.593970268	0.001171796	0.019657675
KCNB1	-1.181947185	0.002787771	0.030831602
TCP10	-2.031339758	0.000758453	0.016486247
TM4SF4	-3.112524456	0.002701434	0.030242329
VIPR1	-1.641817257	0.000553881	0.014663262
FOLH1	-2.562590385	0.000182237	0.010584994
NRTN	-1.006540455	0.002005055	0.026156684
FXYD1	-2.488887573	4.58E-05	0.007487582
RELN	-2.756555698	0.000101357	0.008967806
HOMER2	-1.952942065	0.000155428	0.010188496
XYLB	-1.986337989	0.001588103	0.023076663
NR2F6	-1.577784536	0.000201973	0.011049475
FGB	-2.497008026	0.002262874	0.027647421
GPD1	-2.363563489	0.00152018	0.022654017
SLC17A1	-2.075669855	0.000598461	0.015063312
HP	-1.223527958	0.002362287	0.028170825
NR1I3	-3.091706371	0.000414055	0.013005203

GLUD1	-1.868152842	9.68E-05	0.008856782
IGFALS	-1.807056479	1.36E-05	0.004539536
GRB7	-1.639147406	0.000717644	0.016240629
GCHFR	-2.249288385	0.000822924	0.017073994
PPP1R3C	-1.716424518	0.002438166	0.028657221
NR1H4	-2.73179791	0.000927422	0.017880184
EGFR	-2.273288167	0.00081308	0.016990633
RORC	-2.938774291	0.000198253	0.011049198
SLC15A1	-2.989917818	0.001161335	0.019546423
SCARB1	-1.3311602	0.002973026	0.032097987
HMGCS2	-2.224098156	0.00340891	0.034565756
DEFB1	-2.316700914	0.000797228	0.016779595
COX6A2	-1.06349523	0.002823019	0.031069951
LPA	-3.115770355	0.001133504	0.019288835
ABCB6	-1.469129963	0.001928158	0.0255951
TOM1L1	-1.986842932	0.000189476	0.0107454
SHMT2	-1.147091989	0.003347664	0.034238845
SULT1E1	-2.422336715	0.000172553	0.010584994
ALDH6A1	-2.140853669	0.000385097	0.012777971
RCL1	-2.089477944	0.001797154	0.024608118
GPA33	-1.201869797	0.004684858	0.041866243
GNE	-1.760591393	0.000146752	0.010072696
INHBC	-3.061463074	0.001745159	0.024320249
GLYAT	-3.425651181	0.000183908	0.010584994
TDO2	-3.299958126	0.00110152	0.019128638
ACSM3	-1.725859524	0.00098337	0.018401912
SMO	-1.723664869	6.25E-05	0.008053145
CFHR2	-3.80314201	0.000601763	0.015063312
NET1	-1.528720495	0.000424266	0.013045227
SLC17A2	-1.952522965	0.000318651	0.012254849
TBL1X	-1.10180444	0.005817027	0.047906583
HRSP12	-2.599211738	0.002758089	0.030619436
SLC23A1	-2.450441006	0.000181925	0.010584994
TOB1	-1.662531321	0.001989458	0.026043337
IDH1	-1.089951701	0.002440234	0.028665031
MT1H	-3.06483573	0.000270818	0.011960837
LIPG	-2.172456504	0.005232973	0.044929553
MT2A	-1.476311748	0.002169286	0.027171364
MTHFD1	-1.621740098	0.005552952	0.046619426
ST3GAL6	-1.866823032	0.000273008	0.011960837
NRAP	-2.693770132	0.000765083	0.016486247
MT1F	-2.266628465	4.02E-05	0.007183485
MT1A	-2.478032894	0.000209329	0.011187576
TFPI	-1.262712497	0.003025059	0.032488295

ECI2	-1.092827823	0.003927747	0.037950251
ACAA2	-1.978939971	0.003202909	0.033516452
ZBTB16	-1.744511711	1.81E-05	0.005345652
AGFG2	-1.002837726	0.002882249	0.031517941
SERPINA4	-1.742466866	2.27E-05	0.006099526
PGRMC2	-1.021623627	0.000445745	0.01332889
FST	-3.079812152	0.001538364	0.02272594
MTHFS	-2.221195719	0.000510305	0.014117067
PHYH	-2.231741204	0.000193875	0.010934129
IQGAP2	-1.430365145	0.000748123	0.01645871
SAA4	-2.534697334	0.001018151	0.018559436
MSMO1	-2.593450617	0.000332388	0.012254849
AVIL	-1.432526953	0.001917599	0.025504697
SLC20A2	-1.164000359	0.002490263	0.029052114
MARCO	-1.808178894	0.003306666	0.034045019
SLC16A2	-2.623821843	0.000956711	0.018117246
PPP1R1A	-1.063994941	0.002725429	0.030422285
SLCO1B1	-3.524040668	0.000417038	0.013005203
ILVBL	-1.422265355	0.00139102	0.021612679
COLEC10	-2.54727926	0.003603412	0.035740345
POSTN	-2.539006214	3.64E-05	0.007011546
RBP4	-2.058042639	0.003439585	0.034729259
MASP2	-2.264654446	0.002590941	0.029584257
SLC17A3	-2.709036963	0.004225981	0.039504411
FTCD	-3.187492543	0.000122095	0.009595296
STARD10	-2.14704201	0.000562088	0.014710456
UGP2	-1.461173188	0.001912587	0.025475861
SPP2	-2.759028374	0.001984222	0.026002052
SLC38A3	-1.340311019	0.000512	0.014125672
FMO3	-3.383527293	0.000586652	0.014934015
SDS	-2.876380949	0.000297064	0.012102504
SLC19A2	-1.998677657	0.001523777	0.02265869
ANXA10	-3.330997818	0.001217139	0.02010411
PTGR1	-2.678197908	0.001253023	0.020398568
SARDH	-1.760806585	0.002283522	0.027739133
PKP3	-1.142353892	0.003687717	0.036147402
DLEC1	-1.464510912	0.003661206	0.03607489
TREH	-1.766230452	0.001521914	0.02265869
MME	-1.61516519	0.003712484	0.036352071
AKR7A3	-2.951656361	0.000357749	0.012421423
ALDH1L1	-3.699080393	7.22E-05	0.008374085
PEMT	-2.203782055	0.001003185	0.018484678
GLUD2	-1.965225103	0.000178359	0.010584994
ACOX1	-2.088565194	0.002239522	0.027460704

GRHPR	-1.51130226	0.004882428	0.042898746
MME	-2.189284955	3.23E-06	0.00313707
ATF5	-1.668700713	0.001537802	0.02272594
SLC25A10	-1.372668466	0.001218103	0.02010411
EPB41L1	-1.470625718	0.003025091	0.032488295
SLC27A5	-2.471096365	0.000271324	0.011960837
TPSG1	-3.613013952	1.53E-07	0.001562193
FBXO2	-1.414505937	0.004637705	0.041692376
HAAO	-2.151089401	0.000253972	0.01169459
DMGDH	-2.283919182	0.000499909	0.014019431
NPC1L1	-1.549992061	0.003079412	0.03279566
SEC14L2	-2.843993218	0.000322366	0.012254849
TMEM176B	-2.176346963	0.000848255	0.01731797
MACROD1	-1.494734723	0.00099666	0.018471549
SHPK	-1.130357205	0.002502785	0.029110756
DNMT3L	-3.171397605	1.99E-06	0.002389087
C11orf54	-1.691722518	0.000970387	0.018242554
CLDN15	-1.545004641	0.004233765	0.039542414
FST	-3.323766742	0.000587522	0.014937546
KLF15	-2.093699721	0.005041287	0.04381563
SLC7A9	-1.991097389	0.002273988	0.027705534
TJP3	-1.858861142	0.001706521	0.023922942
GREM1	-1.231926895	0.005499585	0.046396503
SLC25A15	-3.16317934	0.000474776	0.013629768
MTCH2	-1.10628828	0.002170214	0.027171364
GCAT	-2.044810082	0.001763452	0.02446351
FAM162A	-1.234236257	0.000546571	0.014529686
A1CF	-2.823877195	0.000591568	0.014990032
ANGPTL3	-2.793416073	0.003468286	0.034932668
AMACR	-2.086804134	0.000270137	0.011960837
MDN1	-1.39175278	0.001904433	0.025399147
MTFR1	-1.230257929	0.003839275	0.037338644
RHOD	-2.16642814	0.000907084	0.017877478
CARD10	-1.308875734	0.000332067	0.012254849
COBLL1	-1.884904217	0.001749148	0.02434261
SLITRK3	-3.388725968	3.93E-06	0.003192872
JAKMIP2	-1.311999641	0.00326631	0.033866032
KIAA0664	-1.084934317	0.004506266	0.041034755
SLC35D1	-1.962945966	0.002105837	0.026770096
KIAA0564	-1.30252546	0.002961614	0.032008639
SLC39A14	-1.992388132	0.001082302	0.01901645
CUX2	-2.937949819	0.000214797	0.011335849
RPGRIP1L	-1.65453554	0.005784856	0.0477545
MMACHC	-1.727588996	0.004061053	0.038603569

DAK	-2.540773196	0.000142262	0.009971142
AUTS2	-1.211052406	0.004802969	0.042449101
GET4	-1.200902446	0.000636582	0.015326002
PAMR1	-1.151276845	0.003840672	0.037338644
PVRL3	-1.311518644	0.002316045	0.027876304
ARHGEF26	-1.80094313	0.001257627	0.02042618
ISOC1	-1.408168334	0.006227812	0.049979168
MED23	-1.190441635	0.000641955	0.015383885
AIG1	-1.261680719	0.00618304	0.04980902
AADAT	-2.500953608	0.000810399	0.016951958
DCXR	-2.011219036	0.000506672	0.014054629
ACP6	-1.038253312	0.0060918	0.049372839
NSDHL	-1.069908041	0.001435887	0.021911473
CSAD	-1.438858178	0.004046037	0.03854591
SCCPDH	-1.369246349	0.001996633	0.026096837
SERPINA10	-2.842089729	0.001013725	0.018510462
GDF2	-1.506120355	0.003263874	0.033859379
PIPOX	-2.738658319	0.00058547	0.014934015
RAPGEFL1	-1.174449822	0.005061837	0.043956811
CPB2	-3.236841509	0.000377665	0.0126816
SYT17	-1.713477887	0.001005821	0.018505755
HSD17B14	-1.909974349	1.78E-05	0.005331672
CYP39A1	-2.228139986	0.000662238	0.015612309
SLC6A13	-3.980890417	2.46E-05	0.006193459
CLEC1B	-3.382471979	0.000229851	0.011445469
PCYOX1	-1.130220967	7.91E-05	0.008717971
PRODH	-1.729629934	0.0027719	0.030722647
ADD2	-1.257622206	0.005462743	0.046171415
HAO2	-2.657878049	0.000162474	0.01036584
CYP3A4	-2.4286721	0.000932326	0.01793028
RNF43	-1.805836185	0.000317052	0.012254849
HAO1	-3.222975786	0.000260695	0.011775105
BHMT2	-3.362406121	0.000478714	0.013669124
SEMA4G	-2.794176182	0.001253928	0.020398568
SLC38A4	-2.601086889	0.000770143	0.016486247
ANO1	-2.950939317	3.61E-05	0.007011546
LPPR1	-1.938806835	0.002521957	0.029204923
CDHR2	-2.842451799	0.001376736	0.021488871
FAM82A2	-1.29029556	0.001846359	0.025046686
ETNK2	-2.570018556	0.000544718	0.014499307
SLC47A1	-1.992933417	0.000739604	0.016396702
PARP16	-1.086546717	0.001430128	0.021870778
MOCOS	-1.717898484	0.003267833	0.033866032
STAP2	-1.954350682	0.004478365	0.040876238

TTC38	-1.418885681	0.000915003	0.017877478
LIME1	-1.566242581	0.000382671	0.012777971
ACSM5	-2.480992259	0.000880051	0.017666783
MOSC2	-2.364019763	0.000728639	0.016329187
C4orf19	-2.772861575	0.000327369	0.012254849
PNPO	-1.507305941	0.001819591	0.02483207
GPRC5C	-1.751932902	3.27E-05	0.006889194
BEX1	-1.54932791	0.004727645	0.042040979
B3GAT1	-1.902466197	0.001303221	0.020787074
ACSS2	-1.244454685	0.000397381	0.012854122
TMEM176A	-1.752045783	0.002042843	0.026329981
CHDH	-2.478489123	0.002615846	0.029685999
PECR	-2.390541331	0.001965046	0.025849469
PXMP2	-2.432293021	0.000338767	0.012254849
C19orf80	-2.306394829	9.76E-05	0.008856782
ACOT13	-1.125303814	0.003994006	0.038399232
OGDHL	-3.285197077	0.000465364	0.013533993
ECHDC2	-2.313824154	4.37E-06	0.003192872
FGGY	-1.955801911	0.001312282	0.020881961
MOV10L1	-1.440580738	0.004152343	0.039120551
GNMT	-2.884305512	0.000446526	0.01332889
UNC93A	-2.258715956	0.004356982	0.040140857
APOM	-1.62058545	0.001557616	0.022828632
IL17RB	-2.34181727	0.00521733	0.044850243
ERRFI1	-3.109635145	0.001059713	0.018878801
FAM134B	-1.827647909	0.001452089	0.0220908
NECAB2	-2.245851386	0.001121392	0.019278478
DHTKD1	-2.093434113	0.000338636	0.012254849
SLCO1B3	-3.587537182	0.000166171	0.010479165
FAM20C	-1.503400042	0.002155385	0.027112966
ABCC9	-2.207443117	6.12E-06	0.004030853
GPR126	-1.196897954	0.001207625	0.020026719
CGN	-2.527584244	0.000719155	0.016240629
GPHN	-1.515884958	0.004038233	0.038507502
PITPNM2	-1.421410234	0.000268519	0.011954174
SPHK2	-1.010214816	0.000909299	0.017877478
ABCC9	-2.209363252	0.000608657	0.015117199
PPM1H	-1.395438135	0.002796717	0.030880359
HAMP	-3.078185534	3.41E-05	0.007011546
MST1	-2.367772608	0.001048009	0.018768556
CYP4F11	-2.930650688	0.000202675	0.011049475
GPAM	-2.671262833	0.000350909	0.012360149
UGT2B4	-2.797477792	0.001551468	0.022822466
GBA3	-2.809676148	2.54E-05	0.006254726

ADCY1	-2.431664832	0.000574879	0.014877663
KIAA1161	-1.852485562	0.003606621	0.035744069
AQP9	-3.04364268	0.000244646	0.011672752
AVPI1	-1.19977476	0.000426832	0.013045227
HPR	-1.606770069	0.001689247	0.023827496
CFHR3	-2.951283574	0.001247449	0.020340824
CLDN1	-2.210852727	0.003249639	0.033797574
CROT	-1.659423364	0.001845645	0.025046686
GNAO1	-2.524193079	5.11E-05	0.007824954
BMP5	-1.69962357	0.003168556	0.033298891
DAB1	-2.355570228	0.000132478	0.00971712
PSAT1	-2.450767842	0.001325499	0.020984449
HCN3	-1.654490268	0.004369289	0.040181715
DNAJC12	-2.55266976	0.001300637	0.020786211
GYS2	-3.355568563	0.001026938	0.01857039
NR1I2	-3.249573155	0.000562738	0.014710456
SLC22A3	-1.670374472	0.002990073	0.032230901
TP53INP2	-1.579427287	0.001204944	0.020026719
FGG	-2.834774491	0.005078746	0.044008348
FGA	-2.701827434	0.00117561	0.019694888
PRODH2	-2.964332765	0.000247606	0.01169459
CDHR5	-1.948701956	0.004347087	0.040122117
ABCG8	-1.46010976	0.003827617	0.037282741
SLC17A9	-1.667208499	0.002736965	0.030515587
C11orf24	-1.216007628	0.004763201	0.042188945
GPRC5C	-1.539305341	0.000733141	0.016396702
SFXN1	-1.66625465	0.001064968	0.018917117
MOSC1	-2.651434308	0.000331579	0.012254849
PBLD	-3.48502712	7.99E-05	0.008717971
PC	-2.123924279	0.00141634	0.021775325
FN3K	-1.14392602	0.005487068	0.046329192
C11orf1	-1.49210206	0.002071166	0.026507793
ALDH8A1	-3.388017502	0.000706567	0.016099625
SLC13A3	-1.673614472	0.002220873	0.027380043
FGFR3	-2.379371303	0.002507828	0.029123897
ATP13A3	-1.1200535	0.004719279	0.042018665
ELOVL6	-1.273663468	0.003289457	0.033942283
TTPAL	-1.114771669	0.000854045	0.017349426
AGMAT	-3.271760505	0.000147025	0.010072696
TMEM38A	-1.795956782	5.33E-05	0.007887593
C2orf47	-1.229526729	0.003591331	0.035662712
LPAL2	-2.132414458	0.00222585	0.027391776
C7orf10	-2.449979157	0.000830789	0.01715383
RNF128	-2.53251885	0.003032631	0.032513578

GRTP1	-1.973949463	0.002505894	0.029118002
FBXO17	-1.736729785	0.000759538	0.016486247
DNAJC22	-2.931222313	0.000953708	0.018086189
PPP1R3B	-2.004577871	0.000591933	0.014990032
TRIB1	-1.251359245	0.003689811	0.036147402
PNPLA3	-3.142420639	0.000275462	0.012003271
CFHR5	-2.910077886	0.001266056	0.020497859
SLC19A3	-2.137640007	0.000618159	0.015131243
COL18A1	-1.315940573	0.004303614	0.039919393
APOL5	-1.618354215	0.005518949	0.046463862
PQLC1	-1.471212252	0.000327377	0.012254849
AMN	-1.362434602	7.81E-05	0.008717971
HDHD3	-1.948348036	0.000106926	0.009156966
AGXT2	-2.266296613	0.000846673	0.017311984
AGXT2L1	-3.574416599	0.000186018	0.010667842
FAM83D	-1.025425807	0.003632236	0.035858672
FAHD1	-1.316082864	0.000949953	0.018086189
ESPN	-1.183437243	0.000698003	0.016028594
SLC25A18	-2.998203856	0.002614411	0.029685999
ZNRF1	-1.890302924	0.000408695	0.012996767
TOMM40L	-1.346379256	0.000474785	0.013629768
AGTR1	-1.5071762	0.006120912	0.049460979
ALKBH7	-1.116158898	0.002049987	0.026387285
RBP5	-1.095449357	0.00021838	0.011351772
TTBK1	-1.874828635	6.15E-05	0.008053145
ZNRF3	-1.692824793	0.00084976	0.017327378
RTP3	-2.968735923	0.000713896	0.016176355
INHBE	-2.593241404	0.002626594	0.029721626
ZGPAT	-1.91940609	0.000968537	0.018241367
KIAA1804	-1.393783907	0.003051336	0.032615752
CNDP1	-3.542753899	1.07E-05	0.004370993
CFC1	-1.168240241	0.002411676	0.028493505
CBR4	-1.077089686	0.004646809	0.041737459
NR1I2	-3.099679298	0.000235225	0.011553369
PLA2G12B	-2.982206365	0.001897273	0.025366557
DGAT2	-3.553405779	4.54E-05	0.007487582
ABHD1	-1.3884175	0.000235413	0.011553369
CGNL1	-2.705078111	0.001505416	0.022532557
MT4	-1.869778515	0.003963002	0.038236605
RTKN	-1.53624997	0.001879571	0.025312214
TRIM55	-2.390190565	0.000346985	0.012360149
HSPB9	-1.557665022	0.000277778	0.012003271
APOA5	-2.924325836	0.002289022	0.027739133
EMILIN3	-1.917204803	0.001693139	0.023839402

LEAP2	-3.036408571	0.001801692	0.024653709
ADRA1A	-1.456235541	0.003486478	0.035081292
ENO3	-2.475747567	1.04E-05	0.004370993
MIA2	-2.378968086	0.001526043	0.02265869
SEC14L2	-1.348871947	0.00063959	0.015362193
TMEM205	-1.015550089	0.000773363	0.016532964
PSAT1	-2.488723361	0.002143187	0.027026133
MAL2	-2.547973633	0.004723584	0.042038664
B3GAT1	-1.817207984	0.001132877	0.019288835
MOGAT1	-2.804248883	5.92E-05	0.008053145
PGLYRP2	-2.672514657	0.000520127	0.014177441
UGT2B28	-2.792206827	0.001127526	0.019278478
CYP26A1	-1.38545051	6.57E-05	0.008063947
OLFM2	-3.05504229	0.000109908	0.009156966
TADA1	-1.866660574	0.000308254	0.012234335
ASGR2	-3.197991788	0.001315691	0.020903609
SHROOM1	-1.795489632	0.002898294	0.031608746
PAGE5	-1.777625308	8.38E-05	0.00872223
FN1	-3.105647051	7.48E-05	0.008554709
COL18A1	-1.311311342	0.001735522	0.024235584
ABCA6	-1.817477122	0.002736447	0.030515587
SLC22A9	-2.408216855	0.00082832	0.017151099
MBNL3	-1.471585328	0.003109394	0.032943118
ASGR2	-2.956262238	0.000527691	0.014292776
ACOT12	-2.752988267	0.003684463	0.036147039
MLIP	-2.617079692	0.005267072	0.045105928
CALML6	-1.133004932	0.00146549	0.022211915
C13orf27	-1.423036094	0.003333109	0.034161022
PTPRD	-1.133825536	0.002026271	0.026193636
IL1RAP	-1.895275335	0.004556447	0.041289131
GPT2	-2.259453382	0.004084493	0.038751077
PANK1	-2.049272864	0.006122018	0.049460979
REEP6	-2.684420257	0.000427604	0.013049287
ACMSD	-3.637125121	0.000560968	0.014710456
DEPDC7	-2.455965331	0.002194588	0.027219455
MASP2	-1.583069524	0.00318294	0.03337591
MASP1	-2.905491939	0.000701879	0.016028594
A1CF	-3.114555613	0.000574796	0.014877663
NAPRT1	-1.103795997	0.004577164	0.041403363
LRRC45	-1.069568298	0.003408132	0.034565756
CMBL	-2.010848667	0.000151364	0.01013002
ADAMTS13	-2.35758626	0.000196537	0.011046469
SLC39A11	-1.354930212	0.002272757	0.027705534
RANBP3L	-1.721169402	0.001466672	0.022213331

PRAP1	-2.670389745	0.000768874	0.016486247
UROC1	-2.293028498	0.001202509	0.020024815
MARVELD2	-2.168266508	0.003905742	0.0378451
HFE2	-3.038402954	0.000253057	0.01169459
SHMT1	-1.882884489	0.000719914	0.016240629
CES5A	-1.35599214	0.003371023	0.034394209
KLHDC7A	-2.028871277	0.002027133	0.026193636
IL27	-1.22335406	0.002984094	0.032200457
PEMT	-1.614478231	0.001208678	0.020026719
GPR125	-2.149513078	0.000897706	0.017793745
GSTZ1	-2.116843554	0.00106557	0.018917117
GSTA1	-3.206639758	4.08E-05	0.007183485
GSTZ1	-1.958989559	0.000353222	0.012360149
ANKS4B	-1.287707377	0.003398118	0.034549794
ATP1A3	-1.190755472	0.002846281	0.03125108
OCIAD2	-1.552036644	0.004203483	0.039420446
CYB5A	-1.166582304	0.001605154	0.023192377
CLRN3	-2.758592743	0.000292671	0.012095493
FBXO17	-1.68616977	0.001028458	0.01857907
ACOT4	-1.620992206	0.002503844	0.029110756
ZNF385B	-1.679345384	0.00028385	0.012019591
METTL7B	-2.561064327	0.005565367	0.0466569
HIBADH	-1.012993638	0.00122975	0.020263577
PANK1	-2.554385811	0.002620543	0.029695771
AMDHD1	-2.942143613	0.001702966	0.023895361
USP43	-1.182174529	0.003312108	0.034072833
SH3RF2	-1.473511114	0.002921838	0.031739963
SOX5	-1.274870261	0.001265473	0.020497859
NAGS	-2.94417516	3.59E-06	0.003190249
SLC22A1	-3.183009533	6.18E-05	0.008053145
RGN	-1.763917326	0.001682013	0.023755519
SLC22A7	-2.766114894	0.000503756	0.014030957
IL17RC	-1.140683119	0.00039502	0.012854122
SLC23A1	-1.682618859	0.002897445	0.031608746
TTC39C	-1.480843475	0.002562617	0.029497124
FOXA2	-2.155138686	0.005454117	0.046127282
GSTA5	-2.667999899	0.003261637	0.033853372
ALDH5A1	-1.788617176	0.001035729	0.018646769
KCNE2	-1.277461583	0.003409847	0.034565756
LDHD	-2.008684197	0.002201791	0.02724349
TMPRSS6	-2.863201175	0.000426477	0.013045227
ALDH8A1	-3.495121639	0.000420339	0.013042003
FOLH1B	-2.503580659	0.000228486	0.011445469
RDH10	-1.69356817	0.004325866	0.040049374

ALDH4A1	-2.481717071	0.001137054	0.019312891
FCN3	-2.975245321	0.000680193	0.015907016
SLC39A5	-1.62825271	0.000251177	0.01169459
TPPP2	-3.117890447	0.000133268	0.00971712
RNF152	-1.392399554	0.004397994	0.040372954
TMEM86B	-1.207567572	0.000562509	0.014710456
KRTCAP3	-1.319025615	0.001947631	0.025686587
UNC5CL	-1.435492085	0.002191273	0.027219455
PCSK9	-2.143574014	0.001841776	0.025034422
SLC13A5	-3.046885493	0.000758314	0.016486247
GGTLC1	-2.09409535	0.000333562	0.012254849
C11orf35	-1.611655503	0.002138805	0.026995504
SEC14L4	-2.891166332	5.28E-05	0.007863953
GPLD1	-2.473874393	0.000498199	0.014019431
HNF4A	-3.104951191	0.00028957	0.012095493
SLCO4C1	-1.414731836	0.003683922	0.036147039
PIGW	-1.008738837	0.006194444	0.049844697
KLB	-1.745034832	0.000363722	0.012564729
MT1E	-2.069023983	0.00037409	0.012603013
CMTM8	-2.200513586	0.002638238	0.029781319
SERINC2	-2.326486324	0.001993094	0.026067265
SERINC5	-1.579103305	0.000279936	0.012003271
GGTLC1	-1.094180504	0.006196407	0.049844697
FAM151A	-2.470906624	1.98E-05	0.005519794
HSD17B13	-3.564205343	0.00018347	0.010584994
IL6R	-1.460894512	0.004020119	0.03844196
MOGAT3	-1.558717034	0.00343176	0.034683141
HNF4A	-2.25468689	0.001112386	0.019243112
ID1	-1.552241987	0.003137874	0.033124526
HSD11B1	-2.936166331	0.001020748	0.018564781
SLC9B2	-2.202813541	0.002398434	0.02836989
ACSM2B	-2.958169779	0.000208373	0.011165731
STEAP3	-2.828209176	0.002930228	0.031787209
TRIM55	-1.475105814	0.000259422	0.01174361
CERS2	-1.220542467	0.000984897	0.018413405
LDHD	-1.590060271	0.003295817	0.033966377
AADAT	-2.202194019	0.002357254	0.028151978
HPN	-2.680510133	0.00148756	0.022363784
OSGIN1	-1.514822465	0.000437045	0.013140968
TRIM55	-1.768096013	2.54E-05	0.006254726
CCNB1IP1	-1.001996185	0.001556077	0.022822466
C10orf125	-1.550063554	0.00411512	0.038913524
NRAP	-1.849035744	0.002257949	0.027603763
TRIM73	-1.248170059	0.005186178	0.044694394

NNT	-1.316766349	0.002614354	0.029685999
RNF128	-3.30548814	0.001226068	0.020219222
INSIG1	-2.365817884	0.005428676	0.046043942
ALAS1	-2.303374071	0.002656367	0.029946098
PHLPP1	-1.546014438	0.004395992	0.040372727
KRTAP12-4	-1.094956846	0.003897419	0.037800336
ZDHHC16	-1.286862025	0.001001291	0.018471549
TMEM205	-1.33334781	0.005360104	0.04559662
GPR126	-1.4823746	0.000322032	0.012254849
NUDT6	-1.376382556	0.001331737	0.021029389
DHRS4L2	-1.089268955	0.000499308	0.014019431
TJP2	-1.788842252	0.000324562	0.012254849
BDH1	-2.239274144	0.00405296	0.038577987
LSR	-1.678332135	0.000281695	0.012006454
ACSM3	-3.044907607	0.000154213	0.010188496
ABHD15	-1.134000169	0.004086553	0.038751077
DPPA3	-1.113252066	0.004183399	0.039304317
PRB1	-1.137156669	0.000416941	0.013005203
FAM171A2	-1.163311003	0.001014371	0.018510462
CLEC4G	-2.758062635	0.00035679	0.01240923
GOLT1A	-2.224411287	0.004761379	0.042188945
C1S	-1.161093941	0.000991461	0.018468674
GGTLC2	-1.860639001	0.001051491	0.018814407
HOMER2	-1.635210878	0.000305836	0.012195229
GLYAT	-1.166982105	0.003510836	0.03525688
CLYBL	-1.622497979	0.000847114	0.017311984
COLEC11	-2.432511537	0.003165927	0.033298891
RCAN1	-1.512705429	0.005305506	0.045267822
HSPD1	-1.058366553	0.003630533	0.035858672
RCAN1	-1.715102895	0.005473313	0.046232174
LPPR1	-1.998779655	0.002461779	0.028868277
AK4	-2.835481017	0.000347845	0.012360149
CRIP3	-2.676415053	0.000359253	0.012443221
SLC28A1	-1.401999122	0.002306671	0.027873334
ADAMTSL3	-1.481401141	0.000383424	0.012777971
AMACR	-2.4462936	0.001416901	0.021775325
CHCHD10	-1.021900575	0.006068701	0.049259401
GBP7	-2.649235297	0.002555037	0.029454336
ECI2	-1.730166667	0.003767366	0.036830235
SLC26A1	-1.507309116	8.98E-05	0.00872223
HFE2	-3.761654314	0.000434725	0.013136157
USH2A	-4.299344911	6.77E-07	0.001973632
SLC25A47	-3.190045088	0.002303436	0.027873334
FN1	-2.77320186	0.000569534	0.014812232

FGFR4	-1.477916796	0.000233463	0.011513009
FTCD	-1.096721436	0.002813763	0.031010118
HFE2	-3.457602977	0.000432286	0.013136157
ACVRL1	1.511093145	0.001347114	0.021139649
CD3G	1.435709424	0.002284419	0.027739133
CDKN1C	1.517913649	0.004075408	0.038717327
COL1A2	1.213423659	0.002390579	0.02835452
CYP1B1	1.61671274	0.001417629	0.021775325
ENG	1.093639031	0.006041703	0.049122826
IL2RG	1.550878684	0.001291896	0.02070278
ITGB2	1.603350744	0.000261986	0.011799544
JAG1	1.509194262	5.21E-05	0.007824954
JAK3	1.886122465	0.000353562	0.012360149
KIT	1.635295343	0.004736625	0.042063046
LIPA	1.575435698	2.80E-05	0.006427112
MYO5A	1.038544628	0.002896593	0.031608746
PFKM	1.095526447	0.002463433	0.028871094
TIMP3	1.142003223	0.002139436	0.026995504
WAS	1.951685681	0.000476922	0.013647112
CHM	1.140754833	0.001692694	0.023839402
CSF2RB	1.675487771	0.000183676	0.010584994
CYBB	1.530085599	0.002201693	0.02724349
GPD2	1.490563569	1.94E-05	0.00550707
IFNGR1	1.438585002	0.000154608	0.010188496
NCF2	1.667643243	0.000370024	0.012603013
NOTCH3	1.795912453	0.004889173	0.04291374
RFX5	1.466373612	0.000404855	0.01295535
AK1	1.207639199	0.000925599	0.017880184
ATRX	1.47725776	0.000397914	0.012854122
C1QB	1.576643729	0.00034922	0.012360149
HBB	1.314632792	0.002739599	0.030515587
VWF	2.843952784	0.000124745	0.009595296
CD53	2.622729274	6.45E-06	0.004112796
FCGR3A	1.430836112	0.005074627	0.044008348
FCGR3B	2.229453746	0.000253561	0.01169459
CCR5	1.637899088	0.00083213	0.01715383
TAP1	1.348287799	0.000461136	0.013481053
NOS3	2.654090904	0.000739683	0.016396702
BCL2	2.390466847	0.000182684	0.010584994
SELL	2.324686499	0.000336203	0.012254849
ADRA2A	2.056976898	0.000171582	0.010584994
ANXA1	1.142925614	0.003501729	0.035182725
BLVRA	2.349921279	0.000547576	0.014537478
CD3D	1.454834776	0.002350888	0.028117005

CSF2	1.458098203	0.00034643	0.012360149
GNRH1	1.104187487	0.000295734	0.012102504
GSTP1	1.209790063	0.001861644	0.025170418
GUCY1A3	1.483138873	0.000227665	0.011445469
IFNAR2	1.005289622	0.00232159	0.027880924
IL2RB	1.447236891	0.00207829	0.026552173
KCNJ5	2.341391086	0.00093747	0.017971251
KCNJ2	1.430161849	0.005323701	0.045335156
NPR3	2.122990861	1.29E-06	0.002207111
NPY1R	2.43830577	6.91E-05	0.008226635
PAM	1.404558141	0.000992595	0.018471549
PDE1B	1.33028108	0.004298836	0.039893195
PTGER4	1.951091826	0.000946965	0.018068448
PTGS1	1.127880269	0.001914219	0.025476329
ZDHHC13	1.860723208	5.91E-05	0.008053145
PDE9A	1.968451784	0.000592523	0.014990032
RUNX1	1.253262423	0.000370698	0.012603013
GPM6B	1.631698693	0.004208436	0.039431204
WLS	1.593018834	3.59E-05	0.007011546
BICD1	1.896238712	0.001682527	0.023755519
OSBPL8	1.084832175	0.000957509	0.018117246
RBMS3	1.695771739	1.07E-06	0.002207111
RBMS3	1.511945061	0.000395645	0.012854122
TARP	1.745678434	0.001651751	0.023581927
TRIM6	1.692897166	0.000112019	0.009156966
EVI2A	2.08861916	9.07E-05	0.00872223
FCRL6	1.75149917	0.002745012	0.030520361
NCK2	1.471765275	0.001844698	0.025046686
OR5AK2	1.698114763	0.003452838	0.03481143
DNM2	1.060042177	0.005184797	0.044694394
ACTR2	1.086190475	0.006150712	0.04961396
WBP5	1.992561467	1.13E-05	0.004370993
GTDC1	1.30611321	7.14E-05	0.008325379
TCEAL1	1.330245002	0.000193557	0.010934129
CR2	2.372963556	0.003224152	0.033645663
RPS6KA1	1.149513473	0.002002469	0.026139644
RPS6KA2	2.065790389	0.002693772	0.030242329
TCEAL3	1.876834719	0.000182092	0.010584994
ODF2L	1.438476712	0.002578342	0.029538488
SDCBP	1.078982225	0.000319484	0.012254849
SDCBP	1.572045959	0.001001568	0.018471549
ARHGAP25	1.358168431	0.001436009	0.021911473
STH	1.234606701	0.000244707	0.011672752
MXRA7	1.578226422	0.003174477	0.033321403

LGMN	1.737260658	0.000181802	0.010584994
CLSTN1	1.007445499	0.001073662	0.018994698
C20orf194	1.461972043	0.000206658	0.011102961
ARHGAP28	2.658056775	0.003329141	0.034159265
FAM102B	1.146753713	0.004214287	0.039431204
FAM26F	2.588356262	1.54E-05	0.004907082
TMEM44	1.250738915	0.0018883	0.025351442
IL32	1.061121892	0.001465376	0.022211915
SPIN4	1.271042552	0.000951161	0.018086189
TCEAL5	2.097930056	0.000484985	0.013790312
LSP1	1.968454653	0.00055693	0.01467134
C8orf58	1.427292019	0.001208478	0.020026719
BAG5	1.144690726	0.002318673	0.027878698
SAMD3	1.680050018	0.001115631	0.019249515
CARD16	1.648502132	0.000149642	0.010125656
GCOM1	1.402163923	0.000220949	0.011391124
PIR	1.33847182	0.000503658	0.014030957
HIST2H2BF	2.136181115	0.000610233	0.015131243
TGFBR2	1.099383237	0.000606115	0.015107025
CELF2	1.579096051	0.005038916	0.04381563
CD47	1.716507405	0.002023706	0.026193636
CD74	1.782767188	1.69E-06	0.002284534
CD74	1.35535587	0.000222276	0.011408959
CD97	1.164586132	0.004954669	0.043209963
CHI3L2	2.223490262	0.000397823	0.012854122
TSPAN4	1.221045354	0.000511076	0.014119256
CXorf65	2.270401977	0.000273009	0.011960837
ARFIP1	1.323130912	0.000329316	0.012254849
ARHGAP30	2.219537491	0.000303982	0.012168812
PLEKHG1	2.51219561	6.56E-05	0.008063947
CPLX3	2.063126735	0.004354824	0.040139091
SPN	1.286026308	0.000949033	0.018086189
RUNX3	1.60894262	0.004235857	0.039542414
IFIT3	1.282932887	0.000300982	0.012141407
FAM198B	2.294422676	9.30E-07	0.002207111
LYPD5	1.74709691	0.003909647	0.037847012
STXBP1	1.700084303	0.001324139	0.020984449
OAS1	1.517944487	2.94E-05	0.006533081
OAS2	2.061114636	5.24E-07	0.001836216
NPNT	1.944154955	0.000769909	0.016486247
SPECC1	2.58101767	0.001779076	0.024541629
SPECC1	1.791186447	0.002673762	0.030082371
SPECC1	2.71375349	0.000352727	0.012360149
CXCL12	1.280777402	0.00452433	0.04112588

TRAF5	1.727825261	0.000100326	0.008944329
SCN1B	1.661253166	0.000853694	0.017349426
CEP112	1.564774909	0.004879601	0.042898746
PDE4B	1.328483967	0.004496528	0.040977143
CDC42SE1	1.686487096	1.59E-05	0.004926141
EFEMP1	1.53883208	0.003341126	0.034226009
EFEMP1	2.864015208	2.39E-08	0.000487426
MPEG1	2.252026513	0.000366153	0.01259222
C1orf38	1.311928963	0.003078496	0.03279566
SEC14L1	1.233538915	0.002323079	0.02788241
C4orf29	1.226568051	0.003260358	0.033853372
CD53	2.399541038	2.43E-05	0.006193459
HVCN1	1.659895444	0.001887465	0.025351442
CKLF	1.127203682	0.00318864	0.033418522
PCDH17	2.84872156	0.000761557	0.016486247
PHF11	1.038080806	0.000969689	0.018242554
CD3D	1.548389191	0.000580776	0.014895881
FAM110A	1.720802768	0.000574249	0.014877663
FILIP1L	1.755400262	0.000751792	0.016486135
TATDN3	1.480107914	0.001755283	0.02441135
SRGAP2	1.48592631	0.001044014	0.018746344
SLA	1.465182827	9.59E-05	0.008828413
TBXAS1	1.514342877	0.002500607	0.029110756
TKT	1.168508092	0.001232462	0.020291887
ZNF493	1.581855997	0.001667024	0.023710535
TP53I11	1.529032005	0.004345185	0.040122117
ZNF436	1.059275341	0.004033651	0.038507502
WIPF1	1.648486035	0.003759275	0.036774964
WTH3DI	1.259248325	0.000913871	0.017877478
VCAM1	2.199997634	0.001176908	0.019694888
FAM153B	1.484169823	0.000650591	0.015462707
PRNP	1.180306095	0.004518355	0.041089859
CCDC88C	1.246598823	0.004144855	0.039068028
MYBL1	1.654346442	0.003924025	0.037935958
SHANK3	1.593889006	0.002515112	0.029158733
BEX4	1.577503753	0.003992389	0.038399232
PEAR1	1.281519106	0.001070208	0.018966465
USP6NL	1.040421602	0.002925467	0.031752439
SNX29	1.674578784	0.000113074	0.009156966
UNC119B	1.326560921	0.003582948	0.035645009
LILRB5	2.480895986	0.000110716	0.009156966
GAB3	1.387105609	0.002633718	0.029756496
PRF1	1.786355282	0.002194785	0.027219455
BANK1	1.786147331	0.00358309	0.035645009

MRAS	2.183798929	0.000110126	0.009156966
GIT1	1.336388406	3.68E-05	0.00702022
CCDC102B	1.26919622	0.002134787	0.026986877
GPR34	2.191197513	3.12E-05	0.006699452
GCNT1	1.658411889	0.000165723	0.010479165
GCNT1	1.209551859	0.004241881	0.039572439
GPR18	1.230400292	0.005711693	0.047421687
KIF2A	1.188491484	0.000738258	0.016396702
PRKG1	1.200854601	0.001396032	0.021636005
GPR116	1.863853144	0.000458998	0.013481053
8-Sep	1.45912293	1.24E-05	0.004370993
8-Sep	1.401989806	7.59E-05	0.008555575
TMEM91	1.039296477	0.00023158	0.011475579
IFT74	1.035808721	0.000447078	0.01332889
TINF2	1.030971993	0.004930539	0.043073118
SGCE	1.442214029	0.000561167	0.014710456
SGCE	1.310123531	0.000700369	0.016028594
ICAM2	2.521339502	5.62E-05	0.008027439
ICAM2	1.921103069	0.002295907	0.027801447
MRVI1	1.731656553	0.001813342	0.024763337
PHACTR2	1.779936262	3.58E-05	0.007011546
SNRK	1.055702359	0.001535399	0.022715011
PLEKHB2	1.640963167	6.27E-05	0.008053145
FAM72B	1.403670739	0.002181763	0.027219455
LOC645545	1.017926014	0.00352313	0.03534556
INPP4B	1.343273861	0.001892418	0.025351442
FAM13B	1.251143361	0.002540024	0.02933095
HENMT1	1.450823356	0.000649555	0.015456073
ZNF160	1.673935359	0.000725569	0.016288253
SLC25A36	1.386275958	0.0042521	0.039585438
PTPRM	1.0512545	0.000514	0.014145023
NRXN3	2.269747944	0.000277652	0.012003271
CAP1	1.371348838	0.003615684	0.035781781
C1orf38	1.860310917	0.001485884	0.022362968
LYN	2.149767457	0.000135284	0.0097252
PDE4A	1.416174276	0.002393974	0.02835452
CLIC5	2.955582066	0.001171505	0.019657675
C1QC	1.905862412	7.00E-05	0.008226635
TACC1	1.836190597	0.00059592	0.015038689
PPM1M	1.349286588	0.000275328	0.012003271
ADPRH	1.675642796	0.002013579	0.026193636
FAM178B	1.905656403	0.000618857	0.015131243
SLC7A7	1.708276172	0.001599833	0.023164674
SLC39A10	2.171792092	0.000125745	0.009595296

CYBRD1	1.483650234	0.005651369	0.047073987
CDKN1C	1.642009125	0.004859421	0.042795839
ZBTB1	1.22897336	0.0006952	0.016028594
VCAN	1.435189187	0.002169534	0.027171364
FCGR3A	1.779699628	0.000324164	0.012254849
FCGR3A	2.348579232	3.59E-05	0.007011546
GSN	1.247850625	0.000435371	0.013136157
PHTF2	1.007952548	0.002713516	0.030305881
PSIP1	1.200338799	0.002232993	0.027430074
VGLL4	1.155354773	0.000255272	0.01169459
ARHGEF3	1.575744132	0.000203512	0.011049475
ARHGEF3	1.373659963	0.003166653	0.033298891
LIPA	1.708315197	0.000233088	0.011513009
ACTN1	1.111510306	0.000642	0.015383885
IFI27	1.913728538	3.89E-05	0.00715555
GUCY1A3	1.934922344	0.000448648	0.013349109
MARK3	1.363569276	0.000161028	0.010338219
LRRC32	1.287490064	8.97E-05	0.00872223
HMGB2	1.24435267	0.000417646	0.013005203
TBXAS1	1.510827578	0.002188175	0.027219455
MEF2C	2.127784086	8.90E-05	0.00872223
LEPREL1	1.60767993	0.002030826	0.026224752
GSN	1.096917654	0.004012872	0.038427202
STXBP5	1.00603233	0.005233272	0.044929553
DYSF	1.091813561	0.002394357	0.02835452
GSN	1.159620537	0.000745325	0.016450333
UGT8	1.453827125	0.001156676	0.019516273
DTNA	1.256851294	0.000747929	0.01645871
DNAJA4	1.45851125	0.000507659	0.014062909
GUCY1A3	1.608997165	0.001852341	0.025080302
HMGB2	1.416126532	0.000579624	0.014895881
ENPP2	1.58046201	0.00038518	0.012777971
ZBTB4	1.312464233	0.00120034	0.020015789
CYB561D1	1.030229304	0.005743817	0.047553723
GBP5	1.658532744	0.002145484	0.027038401
GIT2	1.031665569	0.001437723	0.021921252
C1QTNF7	1.561769826	0.002387327	0.028349975
ABLIM2	1.85796768	4.24E-05	0.007335659
GAS7	2.656680805	0.00090152	0.017837372
DYSF	1.112208256	0.005538788	0.046571575
PTPN12	1.211097998	0.000683037	0.015932657
GIT2	1.240155088	0.001007691	0.018505755
FAM60A	1.301157043	0.000236229	0.011565571
CPT1C	1.456580289	0.001279671	0.020587681

FCGR2A	1.448493925	0.000498671	0.014019431
C1orf198	1.614495652	0.001127365	0.019278478
BEST1	1.497498304	0.001664042	0.023691126
DAZAP2	1.289367209	0.006057398	0.049191659
SPATA2	1.322701181	0.004829042	0.042605757
SSPN	1.595561071	0.001183638	0.019775086
ACTA2	2.214960143	0.0003558	0.012395929
FAM111A	1.010390762	0.004042781	0.038532876
LPXN	1.115724655	0.005584144	0.046723725
MYBL1	1.356133861	0.003151331	0.033220352
ITGAV	1.435400208	0.000123674	0.009595296
FAM60A	1.21193591	0.000414819	0.013005203
C19orf38	1.037030948	0.004085317	0.038751077
WDR47	1.143921772	0.00340153	0.034565756
ETS1	1.934707291	0.000311432	0.012234335
FGF13	1.585078555	0.005122077	0.044235333
MYO5A	1.613931235	0.000699858	0.016028594
LUZP1	1.370616622	0.001362177	0.02131051
NHS	1.328702649	0.000903915	0.017853622
TMEM51	1.434889911	0.000126864	0.009595296
SERPINE2	1.300643371	0.003794629	0.037049806
PIK3R5	1.973344534	0.002280293	0.027727494
TNFSF13B	1.247704814	0.001879419	0.025312214
TPM4	1.524083799	0.000251282	0.01169459
TRAFD1	1.232433016	0.000410327	0.013005203
SLFN12L	1.816113174	0.004181652	0.039304317
PLAU	1.879142572	0.000769385	0.016486247
SCRN1	1.589552866	0.002672041	0.030082371
CCR1	2.409687096	9.82E-06	0.004370993
PLCL2	1.01571436	0.002009945	0.026170303
PDE6B	1.474148921	0.001627258	0.023379377
KIAA0226	1.652226902	0.000134497	0.0097252
AP2B1	1.624658495	0.001068364	0.018950239
MX1	1.363284974	0.00492924	0.043073118
ANK2	2.78450837	0.00213176	0.026965312
TNFRSF17	1.593446512	0.005743088	0.047553723
CASP1	2.414702908	4.29E-05	0.007335659
CBR3	1.036629203	0.005743901	0.047553723
CD27	1.96889384	1.28E-05	0.004370993
CCDC154	1.0911933	0.001387041	0.021567279
BTN3A1	1.917705519	0.000112873	0.009156966
BTN3A1	1.770599787	2.39E-05	0.006193459
LOC375190	1.954773116	0.001480736	0.022344068
SEC14L1	1.035399408	0.001107073	0.019186762

PRDM1	1.321015421	0.000320307	0.012254849
LDB2	1.083284805	0.002473508	0.028922764
CD68	1.439556663	0.000111797	0.009156966
DPYSL2	1.480287604	0.000147025	0.010072696
DOK1	1.950389434	0.00024362	0.011672752
ITGAM	1.464657444	3.75E-05	0.007028513
FRZB	2.192739769	0.004349224	0.040123708
IGFBP7	1.342148535	6.24E-05	0.008053145
ANXA5	1.182345024	6.59E-05	0.008063947
BIRC3	1.998261093	0.001774187	0.024504666
CLIC1	1.081457011	0.003280751	0.033897042
FABP5	2.112100338	0.000204039	0.011049475
FYB	2.185361569	6.68E-06	0.004134628
ADRBK1	1.732373229	0.000875562	0.017646068
ALOX5AP	1.774828924	0.000630851	0.015260028
CLIC2	2.351115001	0.000225706	0.011445469
ID4	1.740069029	0.000459174	0.013481053
CX3CR1	2.047468999	0.000352925	0.012360149
DKC1	1.187459066	0.002526749	0.029243826
AIF1	2.109347059	8.44E-05	0.00872223
GSTA4	1.258667809	0.001533486	0.022715011
IL18	1.44496195	0.000927328	0.017880184
ACTG1	1.003377789	0.001540523	0.022741371
CD33	1.492959726	0.00102663	0.01857039
CXCL1	3.487224693	4.92E-05	0.00771961
GTF3C2	1.039929325	0.001463996	0.022211915
ATP6V1E1	1.120048771	0.002065434	0.026507793
CD34	1.493160572	0.003248972	0.033797574
FLII	1.111899049	0.001084512	0.01901645
ACTA2	1.829374499	0.001852522	0.025080302
CDH4	1.405627323	0.004325954	0.040049374
AOAH	1.983938711	0.000920319	0.017877478
CRYAB	1.346579068	0.002859814	0.03130615
EMR1	2.021625255	0.001247512	0.020340824
ETV6	1.147270223	0.002999688	0.032300433
AXL	1.402079078	0.000373279	0.012603013
CAPN2	1.433212693	0.00024848	0.01169459
7-Sep	1.3378666	8.29E-05	0.00872223
C5AR1	1.275573387	0.001924704	0.025565886
CTGF	2.340791066	7.01E-05	0.008226635
EZH1	1.63364527	0.002739766	0.030515587
CD1B	1.656769102	0.000457567	0.013481053
COL4A1	1.558648199	0.003126059	0.033068194
GRB2	1.197371477	0.000613652	0.015131243

HCK	2.473316793	0.000160927	0.010338219
HLA-DOA	2.595534254	9.81E-05	0.008864474
HLA-DOB	1.882425808	0.004768759	0.042219853
FPR3	1.555755094	0.000179021	0.010584994
HLA-DPB1	1.926517264	6.60E-05	0.008063947
CD48	1.767332499	0.001702959	0.023895361
CD79A	2.312709515	0.000824276	0.017084696
CTNNA1	1.18959427	0.000426742	0.013045227
CTSC	1.813256025	9.60E-05	0.008828413
DARC	3.204889294	0.000418516	0.013005203
CD72	2.992564713	1.12E-05	0.004370993
CD52	1.8669251	0.001338407	0.021051553
EFNA5	2.259562194	0.000382972	0.012777971
CEACAM3	1.648271732	0.000102637	0.008993312
FPR1	1.669893463	0.000113924	0.009156966
GNAI2	1.168607227	0.004213966	0.039431204
HK3	1.169189275	0.002249363	0.027531776
TNC	2.080758235	0.001164244	0.019579251
KCNN3	1.979400576	0.000617864	0.015131243
KPNA2	1.344688074	0.00148641	0.022362968
LAMB1	1.008649449	0.000877848	0.017658338
MAP4	1.105824862	0.001981095	0.025993599
MX2	1.715244855	0.005318175	0.045315471
IL3RA	1.308934098	0.002277025	0.027705534
ITGAV	1.411366357	0.000312321	0.012234335
KCNJ10	1.798454493	0.003306783	0.034045019
TNPO1	1.07254664	0.001085137	0.01901645
LYN	2.352366739	0.000107508	0.009156966
MEF2C	1.346551606	0.001511245	0.022576581
MX1	1.377201447	6.41E-05	0.008063947
ITPKB	1.846068741	0.005933555	0.048514002
ME2	1.712713346	0.003839948	0.037338644
MNDA	1.752449742	0.004861067	0.042795839
MRC1	1.290699111	0.000620071	0.015142776
ITGA3	2.020717671	0.001154904	0.019502504
ITPR1	1.44369893	0.001029238	0.01857907
HMOX1	2.013577197	0.000162364	0.01036584
IL7R	1.338297026	0.003258538	0.033853372
KLRD1	1.548511467	0.001534832	0.022715011
PDGFA	2.192133287	0.000555344	0.014663262
PFKP	1.779783902	0.00013292	0.00971712
ITPR3	2.532800657	0.000884997	0.017713823
KLRB1	2.021974609	0.00075485	0.016486247
LDHB	1.1749894	0.00216525	0.027171364

MSN	1.214972473	0.000301739	0.012141407
OAS2	1.186953139	0.005882514	0.048290071
PDE7A	1.408121377	0.000751772	0.016486135
LCP1	1.888082463	0.002125879	0.02692428
LTF	1.247971192	0.00558303	0.046723725
MARCKS	2.160477447	0.000135259	0.0097252
MYH9	1.109727444	0.000606767	0.015107025
P2RX5	1.852833913	0.000617105	0.015131243
PLEK	2.276450499	0.000806844	0.016929618
PRKAR1B	1.404611863	0.000158462	0.01030305
PSMB9	2.146943086	0.000238294	0.011579575
PTPN1	1.269832702	0.00029071	0.012095493
RAC2	1.870556271	0.001904687	0.025399147
SRGN	1.286406744	0.000370289	0.012603013
PTPRC	2.113197043	0.000296358	0.012102504
PTX3	3.337163615	0.000359595	0.012443221
RNASE1	2.074101459	4.89E-05	0.00771961
OAS1	1.023878511	0.005704888	0.047384459
PDGFB	1.260748878	0.001413645	0.021775325
SORT1	1.505352281	0.004732081	0.042040979
PDE6D	1.094253031	0.005991012	0.048846849
CXCL6	2.562265928	3.46E-05	0.007011546
SOD3	1.203396919	0.004424858	0.040510267
STXBP1	1.333229756	0.000627235	0.015227949
RGS2	1.804770633	0.003880267	0.03765892
RGS10	1.807133837	5.95E-05	0.008053145
S100A8	2.011313551	0.001168109	0.019628078
PIK3CG	1.759317615	1.04E-05	0.004370993
MAPK11	1.191755249	0.003012195	0.032383872
RENBP	1.458167348	0.005436383	0.04605016
PRELP	1.763488124	0.003664734	0.036076943
SFRP1	1.242871575	0.003689044	0.036147402
PTPN12	1.550675089	0.000615537	0.015131243
TPM4	1.703017209	7.98E-05	0.008717971
CCL5	1.630193783	0.003195336	0.033454343
TIMP2	1.587836172	0.000339746	0.012254849
TFF3	2.47744076	0.000266059	0.011911963
VASP	1.44713072	0.000294379	0.012102504
ZNF267	1.32304405	0.000896156	0.017780295
TDG	1.607013985	0.000535868	0.0143951
SELPLG	1.409068995	0.002388419	0.028349975
THBS2	1.496149424	0.000119839	0.009519964
VCL	1.633390671	0.000528558	0.014292776
TEAD4	1.437828356	2.31E-06	0.002615405

TRIM21	1.225059325	0.000434459	0.013136157
UCP2	1.691090038	0.001182473	0.019771804
TCF21	3.342375921	5.20E-05	0.007824954
TGFBR3	1.094789253	0.005395517	0.045851259
TGFBR2	1.092192792	0.001676082	0.023730166
TLR1	1.835924235	0.000198953	0.011049198
TYROBP	1.239393865	0.001397054	0.021636005
SLC25A12	1.165880727	0.000134448	0.0097252
SMARCA5	1.632120896	0.000229246	0.011445469
RNASET2	1.255792861	0.003558948	0.035512946
GPR68	1.271317451	0.001248161	0.020340824
DYSF	1.310619362	0.002429205	0.028599867
SNN	1.43183123	5.60E-05	0.008027439
PEA15	1.256958818	0.001000219	0.018471549
NSMAF	1.299234371	0.000262392	0.011799544
CXCR4	1.573302479	0.000889639	0.01771828
CDC7	2.194452094	0.000514102	0.014145023
MAP4K3	1.312637004	0.001093249	0.019064259
ITGA8	3.535763696	0.000755855	0.016486247
B3GALT4	1.558969434	0.000627288	0.015227949
PPM1D	1.029031332	0.002816619	0.031016233
NRP1	1.265335592	0.001210471	0.020026719
RPS6KA4	1.248325754	0.001129362	0.019278478
NDST2	1.013690463	0.002792959	0.030872251
SIGLEC5	1.779565402	0.00206866	0.026507793
IQGAP1	1.799681955	0.000166303	0.010479165
CD84	1.887495032	0.000279852	0.012003271
IFITM1	1.031998489	0.004588424	0.041450121
CTSS	1.076828383	0.00308821	0.032862276
SAP30	1.003727799	0.00201622	0.026193636
FABP3	1.041025954	0.004111119	0.038911729
FGF12	1.763389402	0.004029389	0.038495091
SLC7A7	2.24914389	3.64E-05	0.007011546
GNG11	1.704147892	0.00014529	0.010072696
EFEMP1	2.67296788	1.27E-05	0.004370993
FCER1G	1.462416637	0.000310467	0.012234335
SKAP2	1.936576335	0.00041329	0.013005203
WARS	2.027806955	4.38E-06	0.003192872
GYG1	1.486926859	0.001698707	0.023884852
SERPINB9	2.320271056	1.19E-05	0.004370993
ETV5	1.21138056	0.000997009	0.018471549
P2RY6	3.000988729	0.001345556	0.021135344
UBE2L6	1.608801083	1.14E-05	0.004370993
ATP2A1	1.564039298	0.000394349	0.012854122

KLF4	1.876969218	0.003387	0.034471085
COL12A1	1.936983428	4.04E-05	0.007183485
ERG	1.505535803	0.000424405	0.013045227
GNA14	1.026648315	0.003788807	0.037010658
ARRB1	1.15740358	0.003219368	0.033639691
C3AR1	1.241718211	4.66E-05	0.007487582
DACH1	1.747666138	0.003096227	0.032871847
DUSP8	2.004771999	0.000142384	0.009971142
FRG1	1.1381089	0.002179668	0.027219455
CRABP1	3.136579696	0.000149782	0.010125656
EPHA7	1.638967021	0.002063849	0.026507793
BST1	1.194256386	0.001064356	0.018917117
ASAH1	1.4267586	0.004989243	0.043455798
MEOX1	2.498502966	0.00016097	0.010338219
ITGBL1	1.814301932	0.002321371	0.027880924
CNN2	1.289545051	0.000279317	0.012003271
VCAN	1.753311795	0.000583048	0.014912856
KIF2A	1.233405431	0.002212467	0.02732591
NFKBIE	1.021863646	0.002658167	0.029949851
B4GALT5	1.529702015	0.000211061	0.011243021
KIF5B	1.058917358	0.00179099	0.02460588
SPTLC2	2.141864131	0.000118771	0.009471953
MMP9	1.424275108	0.000865946	0.017493386
AIF1	2.067264714	0.000203657	0.011049475
SPARCL1	1.59278004	0.003539657	0.035410497
GRAP2	1.521313159	0.003381912	0.034436462
DOCK2	2.771719491	7.49E-06	0.004367778
C1orf38	1.797222298	0.002025753	0.026193636
ACTN4	1.17326047	0.003131981	0.033113681
JARID2	1.157173612	0.005274134	0.045147472
MTA2	1.779230154	0.000114676	0.009181261
NRXN3	1.641140654	0.004195484	0.039381607
LPXN	1.485412749	0.001791925	0.02460588
ARHGEF6	1.955444216	0.000125494	0.009595296
SLC4A3	1.162326474	0.003489316	0.035092554
ANXA3	2.034794024	0.002696821	0.030242329
COL8A2	2.054197971	0.00300985	0.032375713
CTNS	1.166369668	0.00052543	0.014288541
ADRBK2	2.331338026	0.000471247	0.013629768
CLEC2B	1.875630758	0.00149151	0.022406672
ETS1	1.133956041	0.005442743	0.04605016
S1PR3	1.243436813	0.003352442	0.034238845
CREB5	1.347833074	0.002958779	0.031995595
PIK3CD	1.019721628	0.00455219	0.041286843

PRKX	1.629448541	0.000423547	0.013045227
CBFA2T3	1.277539419	0.004199827	0.039404262
GMFG	2.386359363	7.54E-05	0.008554709
MECP2	1.229576699	0.001020366	0.018564781
SSPN	1.600161207	0.002900555	0.031616516
GPR34	1.733786942	0.001636074	0.023456526
VAV1	1.634113615	0.00131419	0.020896026
HCLS1	1.26483665	0.00566767	0.047171283
NR5A1	3.351102705	0.005701021	0.047381356
MVP	1.057883702	0.001582113	0.023049488
PIP4K2A	1.848945884	9.10E-05	0.00872223
CBFA2T2	1.070536438	0.00526101	0.045091845
CXCL11	1.924398072	0.000791073	0.016692777
SRC	1.71813117	0.00031368	0.012240716
GPR15	1.309427389	0.005809901	0.047869821
GRK5	1.170877414	0.003179148	0.03335328
PTPN14	1.991003653	0.004896263	0.04293905
GNPDA1	1.555755222	8.34E-05	0.00872223
HLA-E	1.116156812	0.001482243	0.022349687
GEM	1.85675129	0.000787071	0.016664319
SH2B3	1.204811464	0.001937918	0.025641305
ITGAD	1.537607156	0.001669904	0.023716253
PIGK	1.369437978	0.000144188	0.010046894
HSPG2	1.01196489	0.002851689	0.03125108
SLCO2A1	1.193714889	0.001614057	0.023255182
TRAF1	1.614547516	0.000150661	0.01013002
ISLR	1.784363967	0.000934454	0.01793028
LMO2	1.379124373	0.000940579	0.01799706
LCK	1.690015581	0.000536955	0.014398436
IFI27	2.429142069	5.69E-05	0.008053145
IFI16	1.873698222	1.17E-05	0.004370993
LCP2	1.751009012	0.002105328	0.026770096
SLC1A5	1.22683116	0.002563083	0.029497124
ACTR2	1.113703561	0.000371484	0.012603013
MAFB	1.631813963	0.003666629	0.036076943
ABI2	1.270938926	0.001912936	0.025475861
NKG7	1.530813423	0.002878621	0.031495138
STX6	1.490375713	0.000916156	0.017877478
SMC4	1.636676599	0.000574965	0.014877663
S100A11	1.029914799	0.005977645	0.04878452
ARPC1B	1.109150523	0.002889694	0.031565543
PTPRCAP	1.519332138	0.000830567	0.01715383
S100A12	1.998978335	0.001672189	0.023716253
NAALADL1	1.383402516	0.003047075	0.032604344

CD180	1.536766415	0.004645765	0.041737459
LGMN	1.266936634	0.003170702	0.033298891
MICB	1.602699512	0.002358571	0.028151978
SMNDC1	1.246677216	0.001743038	0.024307287
IFNGR2	1.279726514	0.000155285	0.010188496
ARL4C	1.868684197	0.001239379	0.020340164
MRC2	1.749474139	0.000254701	0.01169459
MAP3K1	1.142848976	0.000618495	0.015131243
RNASE6	1.271102491	0.001569011	0.022929794
SDCBP	1.107097545	0.004179091	0.039304317
EFS	1.449295087	0.000424601	0.013045227
MFGE8	1.105316965	0.004247249	0.039572439
PIAS3	1.28726861	0.002608203	0.029665221
CWC27	1.384062088	0.000701847	0.016028594
CAPZA1	1.000488093	0.001769519	0.024504666
SIRPB1	1.882538105	9.25E-05	0.008734636
HLA-DMA	1.59531171	7.83E-05	0.008717971
MPHOSPH6	1.242557419	0.004216715	0.039435845
IGSF6	2.264234185	0.000147715	0.010086148
RGS19	2.27733418	0.000366985	0.01259222
TNFAIP2	1.457940008	0.000238724	0.011579575
CD5L	2.126278276	0.001825099	0.024857388
TUBB4	2.958549389	0.000396337	0.012854122
PPP1R2	1.235342262	0.002034286	0.026252837
FBLN5	2.228524976	0.002853253	0.03125108
TUBA1A	1.266875777	0.000920183	0.017877478
IRF9	1.247024961	0.001932441	0.025605119
TLN1	1.068236134	0.001115273	0.019249515
MERTK	1.408666686	0.001147866	0.019448002
TAX1BP1	1.163504167	0.005846466	0.04805211
SPATA2	1.471622921	0.001874395	0.025292569
PDE4A	1.532320533	0.000302703	0.012141407
LRMP	1.564067036	0.005562822	0.0466569
STK4	1.230401188	0.00046473	0.013533993
TRIM22	2.364725888	4.96E-07	0.001836216
OAS3	1.195583797	0.001970031	0.025881694
HCAR3	2.751799877	0.001290729	0.020700334
NOD1	1.623323638	0.002217083	0.027349832
SERPINE2	1.550495317	0.001011183	0.018505755
PNOC	1.497930605	0.000216142	0.011343834
GZMA	1.751528264	0.000602798	0.015063312
PDGFRA	1.633739203	0.000213193	0.011305342
ENPP2	1.38801596	0.000206474	0.011102961
IFI30	1.111336674	0.000713673	0.016176355

SEMA3C	3.225504164	0.000228326	0.011445469
CXCL13	2.474319884	0.002466986	0.028896154
TYRO3	1.453186325	0.000395918	0.012854122
MCAM	2.273374696	0.000832193	0.01715383
CD226	1.515395797	0.002700394	0.030242329
TESK1	1.633155235	0.002238667	0.027460704
NES	2.103932481	0.002128371	0.02693913
BAIAP2	1.684901743	0.000751642	0.016486135
MAP4K5	1.105347286	0.000489496	0.013879943
CFDP1	1.009084211	0.00077037	0.016486247
SEMA4D	2.250627799	0.00055546	0.014663262
PTPRE	2.719253364	4.08E-06	0.003192872
DNAJB4	1.690524269	0.000964156	0.018192427
CAP1	1.550183845	0.000175075	0.010584994
IFI44	1.417761393	0.00010004	0.008944329
GNAI3	1.276769197	4.15E-05	0.007249745
HCP5	1.813001603	0.000302441	0.012141407
FGL2	2.601865536	0.000101907	0.008967806
NEDD9	1.708743715	0.000151831	0.01013002
LAPTM5	1.628991826	0.000770321	0.016486247
CBX1	1.424554649	0.000527597	0.014292776
BTG2	1.554891792	0.000623568	0.015173737
DYNLT1	1.217810167	0.000351332	0.012360149
LYVE1	1.573064054	0.000777184	0.016576452
RAB31	1.536209537	0.000350325	0.012360149
SLA	1.951555777	2.01E-05	0.005519794
LILRA1	2.775176177	0.000346753	0.012360149
UTRN	1.977466648	6.15E-05	0.008053145
CD86	1.384828395	0.002632704	0.029756496
PRSS23	1.765398769	1.84E-05	0.005361646
PIM2	1.772381066	0.001355808	0.021243424
TLE4	1.564572711	0.001208372	0.020026719
ANGPTL2	2.171252921	0.00076971	0.016486247
GPR182	1.750985643	0.00441304	0.040438344
MYO9A	1.214131533	0.001592172	0.023119329
BTN3A3	1.42477174	0.000464522	0.013533993
BTN3A2	1.492864595	0.000238993	0.011579575
STAT1	1.489993922	0.002743482	0.030520361
CD300A	1.693601934	0.00033884	0.012254849
KLRK1	1.568336451	0.002568543	0.029499358
CIT	2.148453509	0.000137931	0.00984617
SP140	1.409253212	0.001425873	0.021822053
GCA	1.296372356	0.004002002	0.038399232
EPB41L3	1.351563565	0.000821714	0.017073994

PIP5K1C	1.081965539	0.001516628	0.022634116
HNRNPH3	1.178750892	0.000544151	0.014499307
MRAS	1.645850825	0.004629716	0.041638892
MAPRE1	1.058745574	0.002190185	0.027219455
PXDN	1.023081432	0.003213517	0.03361023
DKK3	2.078002359	0.000183378	0.010584994
GPR132	1.521135009	0.000240952	0.011629503
DSE	1.52321346	0.000111076	0.009156966
ARL2BP	1.193157144	0.00031281	0.012234335
RAPGEF5	1.612757572	0.000377007	0.012680341
STAP1	1.866157118	0.000406943	0.012975135
TINF2	1.080802663	0.001502301	0.02250612
PYCARD	1.377581484	0.003124191	0.033065567
PHLDA3	2.057934928	1.35E-06	0.002207111
FLRT2	1.553214104	0.002778214	0.030759227
HTRA2	2.16484984	0.000113643	0.009156966
ANP32D	1.058076407	0.00562022	0.046910225
FHOD1	2.449938401	0.005165956	0.044557732
PYCR2	1.037187761	0.001443429	0.021975426
PILRA	1.333639239	5.98E-05	0.008053145
VCL	1.657692019	8.66E-06	0.004370993
HMHA1	1.443609093	0.002579699	0.029538488
NXT1	1.044190093	0.002310035	0.027873334
SSBP2	1.454089846	0.001888702	0.025351442
ANAPC4	1.2388384	0.001869925	0.025248936
FOXP3	1.228492655	0.005512328	0.046446424
CD5	1.198396896	0.000538108	0.014398436
CBX6	1.688333864	0.000912032	0.017877478
GPR160	1.573818933	0.000559592	0.014710456
RAB3IL1	1.817182378	0.002180564	0.027219455
DAPK2	1.952781459	0.000388206	0.012836406
KLRC4	1.787095365	0.002625009	0.029721626
EVI2A	1.597158555	0.001414772	0.021775325
SIGLEC7	1.362896018	0.000336668	0.012254849
KCNMB4	1.007999757	0.000239351	0.011579575
PIK3R5	1.549978032	0.000518244	0.014145023
TNFAIP8	1.99454426	0.00139782	0.021636005
SACS	2.06385438	0.000447212	0.01332889
BCAR1	1.800519637	0.002434964	0.028636078
TMOD3	1.132247541	0.001161245	0.019546423
VGLL4	1.474406644	0.000343309	0.012360149
HSPB8	2.6338619	0.000187529	0.010712144
SESN1	1.390169773	0.000241588	0.011632712
ARMCX2	2.134905583	0.000911456	0.017877478

ZEB2	1.673958701	0.001418061	0.021775325
APOBEC3C	1.745413153	0.000127743	0.009595296
LMCD1	2.562224026	0.000272319	0.011960837
KIAA0226	1.474633724	0.000338973	0.012254849
DOCK10	1.790393026	0.000196949	0.011046469
SPOCK2	1.1930562	0.002509732	0.029129441
SIGLEC8	1.533234821	5.47E-06	0.003721806
RNF144A	1.538704904	0.00335042	0.034238845
KIAA0247	1.16791367	0.000108738	0.009156966
HOXD4	1.392423362	0.005696805	0.04737514
FAM65B	1.806752926	0.001071582	0.01897434
SDC3	1.199963766	0.00112917	0.019278478
ARNT2	1.11601801	0.004658535	0.041771995
AAK1	1.260350708	0.003645612	0.035955951
KIAA0753	1.292027097	0.00473155	0.042040979
RABGAP1L	1.390087769	0.000373571	0.012603013
CHSY1	1.084044817	0.001355649	0.021243424
WDFY3	1.243363766	0.001424995	0.021822053
KIAA0355	1.435141379	0.000653111	0.015472375
PHACTR2	1.853017982	7.90E-05	0.008717971
DENND4B	1.740740008	0.000456611	0.013481053
ARHGAP25	2.314532831	0.000454154	0.013437682
SETX	1.201540071	0.000399433	0.012857899
ENDOD1	2.309830451	5.75E-05	0.008053145
CSTF2T	1.143590985	0.000430862	0.013129084
GPD1L	1.361839691	0.004137371	0.039033531
RRS1	1.097874412	0.00465192	0.041764993
TBC1D4	1.001804579	0.001789086	0.02460588
SEL1L3	1.155039362	0.003157973	0.033267894
CAMTA1	2.354519439	0.005281502	0.045172661
GLS	1.934644127	6.89E-06	0.00413815
6-Sep	1.764415976	0.000764657	0.016486247
TBC1D9	1.08654878	0.000800156	0.016823871
SASH1	1.607536167	0.000454034	0.013437682
ZBTB39	1.222111349	0.000608166	0.015117199
C2CD2	1.956392985	0.001625164	0.023365742
CEP68	1.171638768	0.002275786	0.027705534
MAU2	1.11446827	0.00295884	0.031995595
SWAP70	2.328361916	0.000248303	0.01169459
VPS8	1.317170727	0.003030467	0.032511826
FNBP4	1.269427602	0.001237992	0.020333747
SRGAP2	1.586312466	0.003667408	0.036076943
RGL1	1.333554294	0.001579126	0.023049488
DENND5A	1.210518389	0.000657439	0.015535045

LEPROTL1	1.5596213	0.00124252	0.020340824
DSTYK	1.2256742	0.000180175	0.010584994
NFASC	2.614277445	2.86E-05	0.006479724
PPWD1	1.045097299	0.000697197	0.016028594
PTPN23	1.264103989	0.000129811	0.009694521
RAB11FIP5	2.233538882	0.000321552	0.012254849
DNAJC9	1.487550016	1.59E-05	0.004926141
PARM1	2.360259941	0.003546324	0.035438933
ARAP2	1.574425537	0.000764444	0.016486247
PHF15	1.30475006	0.000461272	0.013481053
C15orf39	1.609364547	0.000160364	0.010338219
TES	1.793826081	9.62E-06	0.004370993
DENND2A	1.471750463	3.25E-05	0.006889194
EP400	1.119235739	0.002905563	0.031648593
GIMAP2	1.659264051	0.000697768	0.016028594
DFNB31	2.146450616	8.68E-05	0.00872223
SIPA1L1	1.148647654	0.001711943	0.023955466
PPP1R16B	1.071313452	0.004190648	0.039354314
LY96	1.968179289	1.51E-05	0.004903772
DKK3	2.718529795	2.72E-05	0.006427112
RHD	1.165735432	0.002569052	0.029499358
MOXD1	1.658825371	1.40E-05	0.004612159
CHST15	1.62943178	0.000287052	0.012083406
RIMBP3	1.694584134	0.003541602	0.035410497
PALLD	2.655724976	8.42E-05	0.00872223
NUB1	1.403132924	0.001774902	0.024504666
ZNF107	1.001044988	0.003498961	0.03517222
ARRDC2	1.102171224	0.003654244	0.036023678
RARB	1.277049258	0.005891497	0.048306259
COMMD10	1.239793103	0.001057379	0.018853665
WSB1	1.091701766	0.004127993	0.038999125
C1QA	1.577758075	8.89E-05	0.00872223
KIAA1279	1.337459167	0.000293911	0.012102504
HDGFRP3	1.249867647	0.001674146	0.023719197
BIN2	2.024605632	0.001506512	0.022532557
UBR5	1.166753011	0.004497932	0.040977143
TAOK2	1.077192001	0.004121523	0.038956025
WBP5	1.745369008	1.90E-05	0.005456676
MZB1	1.420310417	0.006105199	0.049412907
ZCCHC17	1.289558527	0.001305074	0.020787074
OAS2	1.801329777	8.43E-06	0.004370993
PLEKHO1	1.840612696	0.000629099	0.015240915
ARID4B	1.144568124	0.000251437	0.01169459
CRIM1	1.256388621	0.000371818	0.012603013

RAB8B	1.398834977	0.000229766	0.011445469
DACT1	1.588510566	0.005752423	0.047604976
GDPD2	3.253207458	0.000459331	0.013481053
NRN1	1.128942548	0.003874308	0.037629817
PLCE1	2.258244752	0.000881177	0.017672005
ABI3	1.249633784	0.002165939	0.027171364
KLHL3	1.201453782	0.004086404	0.038751077
MIER2	1.620132473	0.003035408	0.032513578
KIF21B	1.832680958	0.000132209	0.00971712
GOLM1	1.702904973	2.58E-05	0.006277875
ARMCX3	1.238227228	0.00323678	0.033732572
XAF1	1.024630402	0.003273408	0.03387222
RTEL1	1.411767398	0.005081553	0.044008348
EPDR1	2.053244704	3.03E-06	0.00313707
PCDH12	1.742638896	0.002184448	0.027219455
SAMD9	1.939320288	0.00017814	0.010584994
TMCO3	1.457455885	0.000843134	0.017306885
GALNT7	1.698846237	0.001288174	0.020691862
FAM46C	1.728591246	0.000291699	0.012095493
1-Mar	2.753251267	1.04E-05	0.004370993
DDX60	1.389511656	3.01E-05	0.006611088
ARGLU1	1.212686117	0.000678758	0.015891654
CCDC88A	1.209021602	0.002157697	0.027125337
PARVA	1.213700838	0.000613101	0.015131243
OSBPL10	1.635659729	0.002603887	0.029649171
MED9	1.133205989	0.003077506	0.03279566
SLFN12	1.699986518	0.000368226	0.012603013
TMEM132A	2.066306719	5.62E-05	0.008027439
GPATCH2	1.483051816	0.000653271	0.015472375
RASIP1	1.861255982	0.000219073	0.011351772
LPCAT2	1.314634501	0.000644682	0.01543005
CCDC109B	1.722783025	0.000746921	0.01645871
RNF111	1.017184056	0.005253574	0.045047023
C1orf56	1.099341811	0.002567732	0.029499358
RAB20	1.401557178	0.001582252	0.023049488
PLEKHB2	1.544893749	0.000425833	0.013045227
VPS13C	1.426430129	0.002915443	0.031694188
C9orf167	1.724519828	0.000123317	0.009595296
HERC6	1.093707823	0.003407169	0.034565756
PNRC2	1.102461908	0.001083768	0.01901645
RIF1	1.037874036	0.003350567	0.034238845
NECAP2	1.336888613	0.000711816	0.016176355
FAR2	1.258845304	0.001554609	0.022822466
SMG8	1.030796528	0.000915889	0.017877478

DRAM1	1.369631821	0.000845169	0.017306885
MIS18BP1	1.440085753	0.0032705	0.03387122
PDP1	1.426140037	0.002386094	0.028349975
TMEM206	1.401581698	0.000889721	0.01771828
SOX18	1.428620155	0.001293417	0.020710903
EXOC1	1.041160217	0.005810218	0.047869821
HMG20A	1.023533615	0.00594413	0.048542141
EXOC2	1.747518237	8.66E-05	0.00872223
SLC25A36	1.408319442	0.00494902	0.043197604
C7orf43	1.158904955	0.00187842	0.025312214
BRF2	1.263121256	0.000632421	0.015279901
AJAP1	2.102104636	0.00017976	0.010584994
CSGALNACT2	1.499307191	0.000459958	0.013481053
TMEM165	1.439335489	0.000815849	0.017031055
HLA-F	1.35353279	0.005543149	0.046571575
ARMCX6	1.175165741	0.001632426	0.023437139
ROBO4	1.658813797	0.003331265	0.034159265
GTPBP2	1.23250356	0.001525202	0.02265869
GIMAP5	1.048570892	0.002344159	0.028069412
CPVL	1.854185051	0.000109164	0.009156966
SH3BP1	1.769101033	0.002051167	0.026387285
SGTB	1.12266562	0.000593824	0.015004334
EAF2	1.943558988	6.57E-05	0.008063947
RIN2	1.874429855	3.06E-05	0.006646822
BATF3	1.037137245	0.004654741	0.041771953
HLA-DRA	1.393132822	0.00050353	0.014030957
EXOC6	1.093901897	0.002835595	0.031157969
HOXA5	1.480236676	0.004678844	0.041866243
ZMIZ1	1.659878016	0.000280957	0.012003271
ZDHHC13	1.385883681	0.000217536	0.011351772
DUSP22	1.23026423	0.004141769	0.039056981
DISC1	1.419187995	0.000997179	0.018471549
KCTD5	1.199972913	0.001081736	0.01901645
ABCC1	2.092990104	3.78E-06	0.003192872
SPATA6	1.204413141	0.004615034	0.041549015
S100A4	1.251747715	0.001248386	0.020340824
RPRM	1.141382605	0.003550601	0.03546432
HOXD8	3.014324295	1.41E-06	0.002207111
CDC42SE1	1.215371025	0.004405336	0.040404019
SUSD2	1.679920228	0.000184055	0.010584994
SEMA3G	1.980423445	0.00132099	0.020971487
ABCC1	2.455354814	4.78E-05	0.007620507
TSHZ3	1.255887741	0.004573797	0.041391236
GALNTL1	2.05822535	0.003225736	0.033645663

AMHR2	1.465944326	0.003029284	0.032511826
ODF2L	1.641152166	0.000542669	0.014482531
MCOLN1	1.530462569	0.000499203	0.014019431
ZNF304	1.09379178	0.005766541	0.047667333
VANGL2	1.015119109	0.001289367	0.020694747
TAOK1	1.071591601	0.003611173	0.035756695
VPS13C	1.685438103	0.000226209	0.011445469
RNF213	1.879713917	0.000404183	0.012954159
C15orf17	1.472223497	0.001383126	0.021539202
RPTOR	1.794715663	0.00353197	0.035364733
NCEH1	2.140400785	8.82E-06	0.004370993
RNF150	2.081762949	0.000933397	0.01793028
ADAM28	1.472888144	0.001707795	0.023922942
SRGAP1	1.26450123	0.003009531	0.032375713
KIAA1430	1.020809135	0.003588625	0.035662712
ZNF462	1.577655129	0.000922492	0.017880184
ARHGAP23	2.642768633	0.000112895	0.009156966
WDFY4	1.394619119	0.001579777	0.023049488
GABBR1	2.227954444	0.000565818	0.014734361
ZBTB4	1.321314461	0.000537949	0.014398436
PDLIM2	1.459939346	0.005299284	0.045248925
MS4A7	2.28404527	6.68E-05	0.008063947
ZNFX1	1.250980991	0.001941822	0.025665926
RAP2A	1.852062553	6.14E-05	0.008053145
SPINT2	1.598182554	0.000531099	0.01434249
TMSB10	1.521839711	0.001261311	0.020461322
GNB4	1.701574837	0.002594718	0.029610829
ELTD1	2.399592037	9.79E-06	0.004370993
NEFH	1.310305632	0.000583629	0.014912856
NAPB	1.002734532	0.004758975	0.042188113
SLAMF7	2.364774341	8.56E-05	0.00872223
TRIM34	1.219643185	0.002225613	0.027391776
HNRNPH3	1.077120621	0.002380954	0.028310751
ITM2B	1.236611668	0.004689856	0.041866243
SAMSN1	1.990114592	0.000270485	0.011960837
ROBO3	1.741109856	0.001077405	0.01901645
ZNF711	1.220652017	0.002309547	0.027873334
MS4A1	1.203337933	0.00512026	0.044235333
C10orf54	2.045064642	0.000167213	0.010504087
IFI6	1.191538284	0.000857828	0.017357211
AXL	1.433008956	0.000822748	0.017073994
GPSM3	1.550853289	0.00028604	0.012065691
NMNAT1	1.29813385	0.001401812	0.021670549
BTN2A3	2.180206942	0.000108891	0.009156966

GBGT1	1.235219409	0.004404449	0.040404019
FAM111A	1.194296366	0.001738985	0.024267333
HERC4	1.090313777	0.005780822	0.047743228
ACSL4	1.443894502	0.000851876	0.017339878
SUSD1	1.464567822	9.48E-05	0.008797851
RASAL3	1.423839809	0.001110252	0.01922554
IFIH1	1.162895541	0.002589038	0.029580638
HIF3A	3.36482164	7.43E-05	0.008554709
DCLRE1B	1.438921501	0.001151684	0.019496498
CENPH	1.047943651	0.003726675	0.036473538
RTP4	1.481993966	0.000533132	0.014363257
MESDC1	1.513797831	0.00150066	0.02250612
CCDC71	1.043597759	0.004473984	0.040876238
SIGLEC1	1.236506143	0.00327576	0.033879392
RASL11B	2.763951565	8.48E-05	0.00872223
IFI6	1.12492531	0.001078157	0.01901645
GINS3	1.563052458	0.000308124	0.012234335
EFHD2	1.404603935	0.000649097	0.015456073
WT1	2.447970502	0.001791992	0.02460588
ATP8B4	1.232736081	0.001256457	0.020423424
C1orf54	1.338541414	0.00105555	0.0188375
C5orf23	1.595151876	0.000302427	0.012141407
TNFAIP8L2	1.774479528	0.002848616	0.03125108
RNF219	1.041414409	0.005789204	0.0477545
ZSWIM4	1.497152574	3.72E-05	0.007024454
ZNF426	1.253543487	0.002620277	0.029695771
GPR172A	1.474352565	0.000739398	0.016396702
RHBDF2	1.836545944	0.000139315	0.009910264
CCDC102B	1.513449349	2.03E-05	0.005519794
LPCAT1	1.949940515	0.000599079	0.015063312
VEPH1	2.209618777	0.000738082	0.016396702
TMEM43	1.180418305	6.22E-05	0.008053145
MMRN2	1.686820543	6.63E-05	0.008063947
CYBRD1	1.779040665	0.000254009	0.01169459
WT1	2.209282669	0.000503453	0.014030957
LILRA6	1.847741695	5.46E-05	0.007966436
PEAK1	2.876686504	0.0003128	0.012234335
BORA	1.085802608	0.002024056	0.026193636
PSTPIP2	2.38716845	0.000385542	0.012777971
CHODL	2.209494057	0.005463859	0.046171415
GAL3ST4	1.377833269	0.000469136	0.01360493
ZNF430	1.465226822	0.001621303	0.023326653
CEP135	1.146501298	0.002309183	0.027873334
LBH	2.224610145	4.57E-05	0.007487582

C6orf62	1.033695312	0.00188271	0.025334653
WLS	1.738434094	8.77E-05	0.00872223
HSPA12A	1.147888485	0.002101096	0.026759816
TMEM156	1.997786503	9.14E-05	0.00872223
RAI1	2.094097306	0.000125898	0.009595296
SLC38A1	2.249575447	0.00014182	0.009971142
ZBTB46	2.331879403	0.000721166	0.016245481
ZBP1	1.635148195	0.000153308	0.010188496
VOPP1	1.441634253	0.00051822	0.014145023
TRAF3IP3	1.594989033	0.004531882	0.041139575
FAM49A	1.660572	0.00123577	0.020313584
PLEKHO2	2.017746802	0.001325918	0.020984449
PLVAP	2.07161996	0.001235642	0.020313584
TTYH3	1.428452224	2.67E-05	0.006406324
NIPA2	1.032339391	0.000253642	0.01169459
C12orf32	1.422389835	0.001101061	0.019128638
ARID5B	1.441726552	0.000178596	0.010584994
PLXNA1	1.432939673	0.0004722	0.013629768
PITPNM3	2.95064319	0.001864721	0.025195335
NDRG3	1.313419588	0.0060134	0.048990251
SH3BGRL3	1.216043851	0.00074311	0.016426854
RASSF4	1.121649102	0.004212029	0.039431204
PPP1R12B	1.330494327	0.001087861	0.019031509
ANTXR1	1.566421118	8.94E-05	0.00872223
GNGT2	1.343960672	0.001261795	0.020461322
FAR1	2.283304541	0.000180159	0.010584994
TBXAS1	1.718106101	0.000155702	0.010188496
EPPK1	1.851828323	0.004694256	0.041868904
BBS2	1.297901213	0.00321963	0.033639691
HMCN1	1.795244619	0.000277791	0.012003271
CPVL	2.514122237	5.19E-05	0.007824954
CCR6	1.444987746	0.001333507	0.021029389
SEMA6B	2.838228976	0.00071243	0.016176355
CCDC115	1.156168865	0.005040509	0.04381563
HCAR1	1.305461539	0.000629315	0.015240915
RNF135	1.508529921	0.000686201	0.015956133
LRRC8C	1.542616954	0.001502539	0.02250612
AFAP1L2	2.900394478	0.001021172	0.018564781
PPP1R9B	1.34706767	0.001025882	0.01857039
SUCNR1	1.447543648	0.001301174	0.020786211
PDZD4	1.478313545	0.000130109	0.009694521
GATA2	2.497055806	0.000108313	0.009156966
RERG	2.474636537	0.001269303	0.020501658
ACRBP	1.643482835	0.001076992	0.01901645

EFS	1.275726997	0.002422926	0.028599867
ACSS1	1.460190117	0.000500914	0.014028354
AIF1	1.607780372	0.000183996	0.010584994
PARD6G	1.362190805	0.000756851	0.016486247
CARD6	1.289555175	0.003668482	0.036076943
C2orf40	3.119829562	0.000886913	0.01771828
TCEAL3	1.521534191	0.000908411	0.017877478
NLRP1	1.315856166	0.000436986	0.013140968
HK1	1.356185842	0.000281033	0.012003271
HLA-DPA1	1.247162354	0.000327348	0.012254849
FAM129A	2.350285026	7.88E-06	0.004370993
STON2	2.035132625	0.002588697	0.029580638
CASP4	1.066599342	9.22E-05	0.008734636
CARD16	1.716956025	0.001014558	0.018510462
IGSF21	2.660783814	0.000126912	0.009595296
PDGFA	2.838798653	1.75E-05	0.005329347
ZNF101	1.021152537	0.003672496	0.036080643
VPS13A	1.175347409	0.004421879	0.040501155
SSH2	1.480994879	0.000418005	0.013005203
PSIP1	1.15648218	0.000305525	0.012195229
PML	1.50003044	0.002483804	0.028995795
ADAM19	1.514850662	0.002638833	0.029781319
SIGLEC10	2.267079228	7.50E-05	0.008554709
HK1	1.252404036	0.002325097	0.027890239
HK1	1.410012795	0.001656883	0.02362216
GBP4	1.659780823	0.000518152	0.014145023
EPSTI1	1.609977861	0.00010416	0.009087706
CASP1	2.894668913	1.79E-06	0.002284534
TANC1	1.534769295	0.003979066	0.038345116
TRIM47	1.132532953	0.004596855	0.0414969
ARHGAP18	1.196055163	0.004701233	0.041876252
BOC	1.406794756	0.000174746	0.010584994
CASP1	2.047473575	3.82E-05	0.007084621
SIGLEC11	2.150820477	0.001054629	0.0188375
ZNF300	1.598047962	0.000257764	0.011720495
CASP1	2.13644278	5.19E-05	0.007824954
SIGLEC12	2.189093001	0.004327704	0.040049374
CASP7	1.136305217	0.006132698	0.049488207
GZMH	1.788434682	0.001963706	0.025848503
LUZP1	1.438353619	0.003376345	0.03441411
FBXO32	1.472529549	0.000347403	0.012360149
BTN2A1	1.501605005	0.00039992	0.012857899
FMNL2	1.594240116	6.84E-05	0.008218765
CYYR1	2.029640118	0.000318912	0.012254849

VNN2	1.004152825	0.001176187	0.019694888
GIT2	1.460673152	0.001644809	0.023499242
RGS18	1.36127993	0.00262772	0.029721626
PTPN6	1.243863277	0.00202112	0.026193636
COL12A1	2.442464631	0.000291495	0.012095493
9-Mar	1.144656686	0.002195848	0.027219455
FLYWCH2	1.790317756	0.000586502	0.014934015
GBP5	2.650856999	8.37E-05	0.00872223
UPF3B	1.439978219	0.000175053	0.010584994
HSPA12B	2.050137988	0.00057737	0.014895881
C20orf160	3.691463854	0.000433225	0.013136157
PTPRE	2.258121144	0.000125349	0.009595296
VCAM1	2.363358309	0.000469047	0.01360493
TRIM34	1.53243377	0.000904224	0.017853622
CD97	2.443475076	1.21E-06	0.002207111
DACH1	1.581325226	0.002589175	0.029580638
ATM	1.024572519	0.004016693	0.03844196
KCTD12	2.162733207	0.000255527	0.01169459
FAM113B	1.417664334	0.000617234	0.015131243
PTPRC	1.307473255	0.000674685	0.015832616
PAQR8	1.462187936	1.07E-05	0.004370993
TTN	1.118224136	0.002499404	0.029110756
ASNS	1.764892275	0.000774292	0.016535518
GIMAP1	1.793588295	0.002289402	0.027739133
ST5	1.097585623	0.00339442	0.034529391
TLR8	2.340979655	4.06E-05	0.007183485
TM4SF18	2.219790065	0.000338871	0.012254849
CD200R1	1.032856496	0.003528483	0.035364733
BATF2	1.442381105	0.004515611	0.041083202
SESN3	1.603570576	6.66E-05	0.008063947
DOCK11	1.053416408	0.002084379	0.026596677
MSR1	1.446715446	0.004053183	0.038577987
SLFN5	1.947616129	0.00041619	0.013005203
PPM1M	1.440244638	0.0008565	0.017351477
RPIA	1.333198863	0.005211262	0.044850243
STAT1	1.725852447	0.000388563	0.012836406
LRRC25	1.226146974	0.001335818	0.021029389
HENMT1	1.726465049	0.002851744	0.03125108
OSBPL3	1.551104885	0.003604495	0.035740345
APOL2	1.087563871	0.000283693	0.012019591
TRIM11	1.542707936	0.00011013	0.009156966
ZNF439	1.359011157	0.000837697	0.017240341
PSMB9	2.62567961	3.46E-06	0.003190249
ZNF420	1.115176729	0.005756246	0.04761731

C9orf25	1.004022047	0.003541743	0.035410497
CCDC151	1.639530949	2.78E-05	0.006427112
MAX	1.560866185	0.000918872	0.017877478
MAX	1.250137109	0.000492442	0.013924795
SNX6	1.218227403	0.004615653	0.041549015
APOBEC3F	1.255252699	0.005098336	0.04410493
EID2B	1.164903731	0.00013242	0.00971712
IL34	3.10364538	0.004953316	0.043209963
ZNF366	1.380714528	0.000765074	0.016486247
GJD3	2.338626169	9.96E-05	0.008944329
OSBPL3	1.014932745	0.005597515	0.046774385
TPM3	1.09263561	0.001480776	0.022344068
OSBPL7	1.379642374	0.002908155	0.031648593
MS4A4A	1.576355024	4.66E-05	0.007487582
PYHIN1	1.741439067	0.006075249	0.049276254
SAMD3	1.441628166	0.003921013	0.037935958
CHMP4C	1.51204285	0.000157882	0.01029815
MLKL	1.26265079	0.000397527	0.012854122
SPIC	2.232002075	0.000284358	0.012019591
PQLC3	1.294339571	0.000982962	0.018401912
TMC8	1.615185522	0.004329397	0.040049374
C7orf60	1.335048161	0.006180121	0.04980902
SLC35G3	1.79603454	0.002359328	0.028151978
SPATA13	1.385228328	0.002427158	0.028599867
FAM116A	1.56361684	0.000918756	0.017877478
GIMAP7	1.236558667	0.002519675	0.02919506
CCDC112	1.248322931	0.000699685	0.016028594
TES	1.507784858	0.00082177	0.017073994
SEMA3D	3.378289588	9.03E-05	0.00872223
SPECC1	1.050956465	0.006122056	0.049460979
NEDD1	1.230612366	0.001124589	0.019278478
PMP22	1.441663176	1.24E-05	0.004370993
KCNJ1	1.000507526	0.001603344	0.023182626
CSF3R	2.243083136	0.000199163	0.011049198
SH2D3C	1.822701461	0.001832618	0.024943157
SNX16	1.479966893	0.000206272	0.011102961
LACC1	1.236616145	0.002069575	0.026507793
HIF3A	2.838192027	0.001023814	0.018570027
IGLL1	1.755676478	0.000783125	0.016602575
DKFZp667G2110	1.033370443	0.000314172	0.012240716
P2RX2	1.495358265	0.000953302	0.018086189
FYN	1.23358216	0.002232883	0.027430074
LIX1L	1.072724634	0.005593026	0.046761587
MEIS2	1.129837341	0.003838479	0.037338644

ZNF610	1.254528069	5.77E-05	0.008053145
CSF1	1.379363295	0.003254154	0.033827294
PLEKHH2	1.548295419	0.001812187	0.024763337
SLC2A14	1.641938721	0.000602351	0.015063312
CD8B	1.271978291	0.0023698	0.028210989
IL15RA	1.073720306	0.004004717	0.038399232
C1QC	2.076127472	0.000238346	0.011579575
FAM98B	1.420350319	0.000352812	0.012360149
PAPLN	1.769746665	0.000220116	0.011376953
C1orf162	1.322131511	0.001667728	0.023710535
TIGIT	1.768751306	0.000485967	0.013799018
NFATC1	1.529435449	0.001716757	0.024006379
GIMAP8	1.634024307	0.000901654	0.017837372
ATP2A3	1.489919299	0.001951792	0.025708252
ST6GALNAC4	1.047129598	0.004614122	0.041549015
RHOC	1.029178926	0.000635167	0.015309991
CD163L1	2.62405844	0.000647031	0.015431998
DNMT3A	1.037146801	0.001795241	0.02460588
TMTC1	2.859328032	0.002222419	0.027382568
PAN3	1.233858111	0.001153644	0.019497347
GOLM1	1.89417306	0.000393492	0.012854122
P2RY6	1.754158012	0.003583805	0.035645009
P2RY13	1.615329942	0.00186012	0.025166469
ARMCX2	1.864844929	0.000844429	0.017306885
DTX3	1.421545935	0.002194954	0.027219455
MEST	1.57635577	0.002779926	0.030761501
MB21D2	1.091634341	0.003240119	0.033750135
NLRC3	1.688316437	0.001273568	0.020554276
PILRA	1.483680719	8.08E-05	0.00872223
STX12	1.101927375	0.000919845	0.017877478
ASB6	1.018602191	0.002311532	0.027874919
HCAR2	2.639564968	0.002922763	0.031739963
WDFY3	1.488547899	0.003061169	0.032669538
FERMT3	2.380249666	8.08E-05	0.00872223
OGT	1.008814474	0.005266592	0.045105928
NCOA3	1.429086947	0.002935193	0.031824168
OLFML2A	3.195138142	0.005081804	0.044008348
LYPD5	1.290024682	0.000698904	0.016028594
KLC1	1.120971442	0.000404122	0.012954159
IKZF3	1.536251571	0.003768533	0.036830235
MICALL2	1.147319424	0.005988318	0.048844385
LRRC70	1.297291061	0.000214967	0.011335849
ARHGAP30	1.60531513	0.005510996	0.046446424
NPM2	2.22214267	0.00054085	0.014452868

TRNT1	1.329160303	0.000616463	0.015131243
BCL6B	1.703168211	0.000684411	0.015932657
SLC16A9	3.18593365	0.000200702	0.011049475
LILRA5	1.900645133	0.00018784	0.010712144
CD247	1.403107159	0.003924409	0.037935958
HEATR3	1.313650955	0.000921938	0.017880184
GEM	1.676169507	0.001101846	0.019128638
CREB5	1.49767521	0.000222961	0.011408959
CLEC7A	1.27788916	0.005163562	0.044555906
RASSF5	1.304288247	0.001611865	0.023240001
BTN3A3	2.025197543	0.000506102	0.014054629
RNASE1	1.980213605	9.41E-05	0.008797851
PRNP	1.146152425	0.000580233	0.014895881
IKZF3	2.06313235	0.00077937	0.016576452
WDR86	1.710458309	0.004877387	0.042898746
PYHIN1	2.615228782	1.73E-06	0.002284534
INO80C	1.336009445	0.000832653	0.01715383
C2CD2	2.130140553	0.000189004	0.0107454
ZNF468	1.025066676	0.002833494	0.031154826
BTN3A1	1.297158632	0.001420726	0.021775935
RNASE1	2.07221658	9.13E-05	0.00872223
OLFML1	1.11633996	0.001126868	0.019278478
TYROBP	1.447144869	0.00035482	0.012382908
C18orf34	2.059035966	0.000690122	0.016010827
UBA3	1.459985385	0.000337004	0.012254849
SYNJ1	1.07192483	0.005280106	0.045172661
DOCK8	2.511145398	1.18E-05	0.004370993
PMEPA1	1.596398286	0.000334275	0.012254849
TCF21	2.04299546	0.00163861	0.023472434
SPRY1	1.709106179	0.000398688	0.012857899
LST1	1.48983359	0.001565863	0.022916601
MAP3K3	1.269282355	0.002698602	0.030242329
FYB	1.433123073	0.000916728	0.017877478
QKI	1.797502179	0.000484474	0.013790312
MS4A7	1.474492221	0.002833783	0.031154826
SLC24A5	2.129997252	0.000884966	0.017713823
PRMT2	1.198249396	0.003880994	0.03765892
RNF216	1.159645492	0.000978644	0.01836396
FAM110A	1.548061042	0.001523871	0.02265869
DPY19L3	1.29984522	0.00121609	0.020103403
CMPK2	1.472030053	0.001763827	0.02446351
ABCG1	2.022627975	0.00057938	0.014895881
C17orf87	1.52989802	0.00093238	0.01793028
ZC3H12D	1.579741909	0.004246761	0.039572439

TNFAIP8L3	3.23150647	8.99E-05	0.00872223
ABCG1	1.685160362	0.001975034	0.025930736
UAP1L1	2.048084227	0.000352033	0.012360149
CLEC9A	2.192614495	0.000256048	0.01169459
TPM2	2.02297931	0.00175733	0.024423172
MBNL1	1.110255344	0.002612774	0.029685999
CLEC17A	1.299274495	0.003433318	0.034683141
WARS	1.98062938	4.54E-06	0.003192872
C17orf76	3.132010018	0.000867129	0.017493386
NA	1.643247765	0.000267057	0.011930481
miRNA symbol	log2FC	P.Value	FDR
hsa-miR-1228-5p	-3.228350247	0.022395051	0.843052701
hsa-miR-1276	-3.677628249	0.036501016	0.843052701
hsa-miR-1469	-4.705041152	0.003406286	0.843052701
hsa-miR-18a-5p	-1.333305991	0.006902254	0.843052701
hsa-miR-18b-5p	-3.619377623	0.045962111	0.843052701
hsa-miR-19a-3p	-1.149741963	0.014454338	0.843052701
hsa-miR-202-5p	-4.192940057	0.018819048	0.843052701
hsa-miR-203	-1.807312576	0.013739189	0.843052701
hsa-miR-219-5p	-3.11762936	0.03602499	0.843052701
hsa-miR-222-5p	-3.839114679	0.01525356	0.843052701
hsa-miR-2277-5p	-1.199861087	0.00576849	0.843052701
hsa-miR-3144-3p	-3.49389259	0.036984152	0.843052701
hsa-miR-3150a-5p	-3.731765685	0.030399108	0.843052701
hsa-miR-3150b-3p	-3.97710253	0.016657363	0.843052701
hsa-miR-346	-3.59190521	0.045831871	0.843052701
hsa-miR-34c-5p	-1.693761398	0.025868716	0.843052701
hsa-miR-3611	-1.495410563	0.003417465	0.843052701
hsa-miR-3664-3p	-2.008700869	0.038940185	0.843052701
hsa-miR-3925-3p	-3.618634591	0.002884694	0.843052701
hsa-miR-3942-5p	-3.352483165	0.041428783	0.843052701
hsa-miR-4424	-3.308095237	0.024319791	0.843052701
hsa-miR-4482-5p	-2.605695179	0.014138	0.843052701
hsa-miR-4482-5p	-2.605695179	0.014138	0.843052701
hsa-miR-4637	-2.905732563	0.027507717	0.843052701
hsa-miR-4728-3p	-3.470829221	0.042138407	0.843052701
hsa-miR-487a	-3.106245758	0.035532402	0.843052701
hsa-miR-5010-5p	-3.366899459	0.018106845	0.843052701
hsa-miR-508-3p	-3.677151471	0.037117638	0.843052701
hsa-miR-545-3p	-2.950782001	0.014838947	0.843052701
hsa-miR-548ak	-3.339045779	0.025425157	0.843052701
hsa-miR-548ap-5p	-4.160341661	0.01561358	0.843052701
hsa-miR-548d-3p	-3.421760985	0.034371277	0.843052701
hsa-miR-548d-3p	-3.421760985	0.034371277	0.843052701

hsa-miR-548w	-3.827245304	0.011746697	0.843052701
hsa-miR-551b-3p	-3.999937139	0.03297371	0.843052701
hsa-miR-556-3p	-3.102621416	0.048291998	0.843052701
hsa-miR-5698	-2.541399288	0.014266336	0.843052701
hsa-miR-590-5p	-3.088961018	0.029623539	0.843052701
hsa-miR-597	-4.168198237	0.016154285	0.843052701
hsa-miR-600	-3.315866681	0.04565542	0.843052701
hsa-miR-1179	3.627066913	0.012288325	0.843052701
hsa-miR-4326	4.626483445	0.034429194	0.843052701
hsa-miR-4474-3p	3.193854211	0.047349323	0.843052701
hsa-miR-4532	3.941865279	0.022800818	0.843052701
hsa-miR-488-3p	3.472672942	0.034428028	0.843052701
hsa-miR-940	4.268661174	0.007736558	0.843052701
