# Supplementary material for: Coding and non-coding gene regulatory networks underlie the immune response in liver cirrhosis
Source: PLoS One. 2017 Mar 29;12(3):e0174142. doi: 10.1371/journal.pone.0174142 (PMC5371304; doi:10.1371/journal.pone.0174142)
Supplement: S2 Table — (PDF) [file pone.0174142.s002.pdf]

Supplementary Table 2. The function of differentially expressed genes in liver cirrhosis

GO Term	P Value	FDR
GO:0009611~response to wounding	4.64E-26	8.62E-23
GO:0055114~oxidation reduction	2.85E-21	5.29E-18
GO:0009063~cellular amino acid catabolic process	7.50E-16	1.44E-12
GO:0016054~organic acid catabolic process	2.15E-15	3.92E-12
GO:0046395~carboxylic acid catabolic process	2.15E-15	3.92E-12
GO:0006952~defense response	2.92E-15	5.36E-12
GO:0006955~immune response	8.98E-15	1.67E-11
GO:0002526~acute inflammatory response	1.28E-14	2.37E-11
GO:0009310~amine catabolic process	1.83E-14	3.41E-11
GO:0006956~complement activation	2.40E-14	4.46E-11
GO:0002541~activation of plasma proteins involved in acute inflammatory response	5.14E-14	9.55E-11
GO:0042060~wound healing	9.74E-14	1.81E-10
GO:0002253~activation of immune response	2.61E-12	4.86E-09
GO:0002252~immune effector process	5.02E-12	9.33E-09
GO:0016064~immunoglobulin mediated immune response	5.73E-12	1.07E-08
GO:0006954~inflammatory response	5.77E-12	1.07E-08
GO:0019724~B cell mediated immunity	1.64E-11	3.04E-08
GO:0002684~positive regulation of immune system process	2.36E-11	4.38E-08
GO:0050778~positive regulation of immune response	2.55E-11	4.74E-08
GO:0006958~complement activation, classical pathway	3.99E-11	7.41E-08
GO:0008202~steroid metabolic process	1.62E-10	3.02E-07
GO:0002455~humoral immune response mediated by circulating immunoglobulin	1.92E-10	3.57E-07
GO:0045087~innate immune response	2.32E-10	4.32E-07
GO:0051605~protein maturation by peptide bond cleavage	5.12E-10	9.52E-07
GO:0019439~aromatic compound catabolic process	5.83E-10	1.08E-06
GO:0006959~humoral immune response	1.13E-09	2.09E-06
GO:0002449~lymphocyte mediated immunity	1.17E-09	2.18E-06
GO:0048584~positive regulation of response to stimulus	1.19E-09	2.21E-06
GO:0002443~leukocyte mediated immunity	2.41E-09	4.47E-06
GO:0007596~blood coagulation	3.51E-09	6.52E-06
GO:0050817~coagulation	3.51E-09	6.52E-06
GO:0007155~cell adhesion	1.24E-08	2.31E-05
GO:0002460~adaptive immune response based on somatic recombination of immune receptors built from immunoglobulin superfamily domains	1.33E-08	2.46E-05
GO:0002250~adaptive immune response	1.33E-08	2.46E-05
GO:0022610~biological adhesion	1.36E-08	2.53E-05
GO:0007599~hemostasis	1.74E-08	3.24E-05
GO:0006631~fatty acid metabolic process	1.80E-08	3.35E-05

GO:0055088~lipid homeostasis	2.09E-08	3.89E-05
GO:0043603~cellular amide metabolic process	2.71E-08	5.04E-05
GO:0016053~organic acid biosynthetic process	3.56E-08	6.61E-05
GO:0046394~carboxylic acid biosynthetic process	3.56E-08	6.61E-05
GO:0010033~response to organic substance	3.95E-08	7.35E-05
GO:0016485~protein processing	4.68E-08	8.70E-05
GO:0055092~sterol homeostasis	1.08E-07	2.00E-04
GO:0042632~cholesterol homeostasis	1.08E-07	2.00E-04
GO:0051604~protein maturation	1.32E-07	2.46E-04
GO:0006805~xenobiotic metabolic process	1.35E-07	2.50E-04
GO:0006732~coenzyme metabolic process	2.21E-07	4.10E-04
GO:0006006~glucose metabolic process	2.21E-07	4.10E-04
GO:0009719~response to endogenous stimulus	2.33E-07	4.34E-04
GO:0005996~monosaccharide metabolic process	3.15E-07	5.85E-04
GO:0009074~aromatic amino acid family catabolic process	3.21E-07	5.97E-04
GO:0009725~response to hormone stimulus	3.28E-07	6.10E-04
GO:0051270~regulation of cell motion	4.34E-07	8.06E-04
GO:0051241~negative regulation of multicellular organismal process	5.44E-07	0.001011755
GO:0008652~cellular amino acid biosynthetic process	6.37E-07	0.001183402
GO:0009309~amine biosynthetic process	7.47E-07	0.00138879
GO:0001775~cell activation	7.77E-07	0.001443669
GO:0009064~glutamine family amino acid metabolic process	9.02E-07	0.00167699
GO:0009410~response to xenobiotic stimulus	1.06E-06	0.00196748
GO:0006869~lipid transport	1.26E-06	0.002346754
GO:0051186~cofactor metabolic process	1.51E-06	0.002797861
GO:0034367~macromolecular complex remodeling	1.52E-06	0.002818456
GO:0034369~plasma lipoprotein particle remodeling	1.52E-06	0.002818456
GO:0034368~protein-lipid complex remodeling	1.52E-06	0.002818456
GO:0019318~hexose metabolic process	2.43E-06	0.00451838
GO:0046486~glycerolipid metabolic process	2.88E-06	0.005346354
GO:0006928~cell motion	3.22E-06	0.005988764
GO:0030334~regulation of cell migration	3.28E-06	0.006089154
GO:0010876~lipid localization	3.44E-06	0.006396151
GO:0019748~secondary metabolic process	6.21E-06	0.011542888
GO:0034637~cellular carbohydrate biosynthetic process	7.25E-06	0.013468979
GO:0042110~T cell activation	8.27E-06	0.015363864
GO:0019319~hexose biosynthetic process	8.31E-06	0.015439151
GO:0050878~regulation of body fluid levels	1.33E-05	0.024780907
GO:0006575~cellular amino acid derivative metabolic process	1.35E-05	0.025000014
GO:0009065~glutamine family amino acid catabolic process	1.41E-05	0.026255891
GO:0015918~sterol transport	1.55E-05	0.028809328
GO:0030301~cholesterol transport	1.55E-05	0.028809328

GO:0046364~monosaccharide biosynthetic process	1.55E-05	0.028809328
GO:0051260~protein homooligomerization	1.77E-05	0.032862263
GO:0045321~leukocyte activation	2.20E-05	0.040867543
GO:0006094~gluconeogenesis	2.41E-05	0.044737823
GO:0019400~alditol metabolic process	2.41E-05	0.044737823
