# Supplementary material for: Coding and non-coding gene regulatory networks underlie the immune response in liver cirrhosis
Source: PLoS One. 2017 Mar 29;12(3):e0174142. doi: 10.1371/journal.pone.0174142 (PMC5371304; doi:10.1371/journal.pone.0174142)
Supplement: S3 Table — (PDF) [file pone.0174142.s003.pdf]

Supplementary Table 3. The function of overlapping genes between liver cirrhosis and HCC.

GO Term	P Value	FDR
GO:0006955~immune response	1.45E-10	2.47E-07
GO:0002684~positive regulation of immune system process	2.74E-08	4.67E-05
GO:0050870~positive regulation of T cell activation	6.40E-08	1.09E-04
GO:0002696~positive regulation of leukocyte activation	2.59E-07	4.41E-04
GO:0050867~positive regulation of cell activation	4.30E-07	7.33E-04
GO:0050863~regulation of T cell activation	7.63E-07	0.001301745
GO:0051251~positive regulation of lymphocyte activation	8.11E-07	0.001382351
GO:0007155~cell adhesion	3.35E-06	0.00571544
GO:0022610~biological adhesion	3.45E-06	0.005881576
GO:0002495~antigen processing and presentation of peptide antigen via MHC class II	5.59E-06	0.00953355
GO:0019886~antigen processing and presentation of exogenous peptide antigen via MHC class II	5.59E-06	0.00953355
GO:0002694~regulation of leukocyte activation	5.63E-06	0.00960007
GO:0051249~regulation of lymphocyte activation	9.10E-06	0.015523746
GO:0050865~regulation of cell activation	1.00E-05	0.0170682
GO:0019882~antigen processing and presentation	1.20E-05	0.020454123
GO:0030097~hemopoiesis	1.39E-05	0.023624649
GO:0045582~positive regulation of T cell differentiation	1.49E-05	0.025366998
GO:0002521~leukocyte differentiation	1.55E-05	0.026455287
GO:0002520~immune system development	2.24E-05	0.038177493
GO:0045580~regulation of T cell differentiation	2.43E-05	0.041392952
GO:0002714~positive regulation of B cell mediated immunity	2.53E-05	0.043147503
GO:0002478~antigen processing and presentation of exogenous peptide antigen	2.53E-05	0.043147503
GO:0002891~positive regulation of immunoglobulin mediated immune response	2.53E-05	0.043147503
GO:0045621~positive regulation of lymphocyte differentiation	2.55E-05	0.043521229
